# Metallodendrimers Unveiled: Investigating the Formation and Features of Double-Decker Silsesquioxane-Based Silylferrocene Dendrimers

**DOI:** 10.1021/acs.inorgchem.3c02628

**Published:** 2023-09-29

**Authors:** Aleksandra Mrzygłód, M. Pilar García Armada, Monika Rzonsowska, Beata Dudziec, Marek Nowicki

**Affiliations:** † Faculty of Chemistry, Adam Mickiewicz University in Poznan, Uniwersytetu Poznańskiego 8, 61-614 Poznan, Poland; ‡ Centre for Advanced Technologies, Adam Mickiewicz University in Poznan, Uniwersytetu Poznańskiego 10, 61-614 Poznan, Poland; § Departamento de Ingeniería Química y Medio Ambiente, Escuela Técnica Superior de Ingenieros Industriales, Universidad Politécnica de Madrid, José Gutierrez Abascal 2, 28006 Madrid, Spain; ∥ Institute of Physics, Poznan University of Technology, Piotrowo 3, 60-965 Poznan, Poland

## Abstract

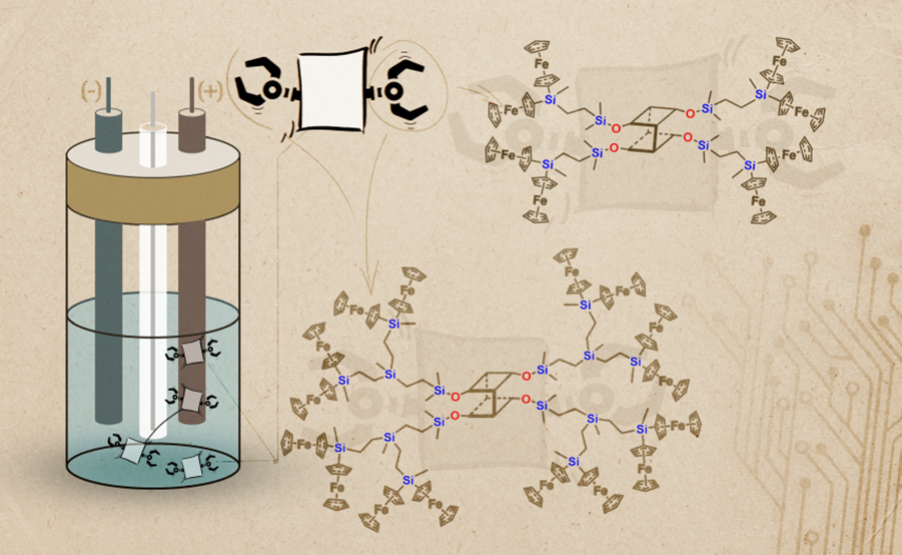

Dendrimers exhibiting reversible redox properties have attracted extensive attention for their potential as electron transfer mediators, catalysts, and molecular sensors. In this study, we introduce intriguing G1 and G2 dendrimers featuring double-decker silsesquioxane cores and silylferrocene moieties. Through a carefully orchestrated sequence of condensation, reduction, and hydrosilylation reactions, these compounds were synthesized and comprehensively characterized spectroscopically and spectrometrically. Our investigation also encompassed the examination of their properties, including thermal stability, solubility in common organic solvents, and electrochemical behavior. We determined that these dendrimers possess the capability to form monolayers on platinum electrodes, which we conclusively demonstrated through the probing of cyclic voltammetry, electrochemical impedance spectroscopy, and scanning electron microscopy imaging. Notably, this study marks the first-ever example of modifying double-decker silsesquioxane cores with ferrocene groups while simultaneously representing one of the scarce instances of dendrimers exhibiting an open double-decker silsesquioxane core.

## Abstract 2

This research focuses on the synthesis of G1 and G2 dendrimers with double-decker silsesquioxane cores and silylferrocene moieties and the verification of their properties, including thermal stability, solubility in common organic solvents, and electrochemical behavior.

## Introduction

Ferrocene, as a representative of a classic organometallic compound, of a sandwich aromatic structure with iron as the central atom, is characterized by undoubtedly impressive redox properties and chemical and thermal stability. Ferrocene-based compounds found many applications from fuel additives, pharmaceuticals, catalysts, capacitors, and porous systems for dyes/heavy ion removal to electrochemical sensors.^1−7^ However, ferrocene itself shows poor adhesion to surfaces. Therefore, its modification using diverse systems from oligo- and polysiloxanes and graphene oxide to dendrimers makes it possible to enhance this and other properties.^2,8−11^

Dendrimers are specific systems with defined, mainly spherical, three-dimensional structures. Their construction can be divided into the following fragments: a multifunctional core possessing extended arms with terminal functional groups that significantly affect the properties of dendrimers.^12^ The arms of the dendritic systems can be built of diverse units such as polyaminoester, carbosilanes, or polyamidoamines.^13−15^ They are characterized by high molecular weights that correspond with a number of electroactive ferrocene terminal groups exhibiting good solubility and adhesion to electrodes.^16^ There are scientific reports on the use of ferrocene dendrimers as electrode modifiers for direct quantification of DEHP [di(2-ethylhexyl)phtalate],^2^ for monitoring of ATP^2–^,^17^ glucose,^18^ boric acid,^19^ oxidation-triggered drug delivery,^20^ and anion recognition,^21^ or as a catalyst.^3,22^

Organosilicon compounds are often used in dendritic systems, e.g., silanes, cyclosiloxanes, 1,3,5-trisilacyclohexane, or more complex structures such as silsesquioxanes.^7,15,23−28^ They are known as polyhedral oligomeric silsesquioxanes (SQs, POSS) and represent a class of hybrid organic–inorganic compounds that have garnered significant attention in the field of materials chemistry. These unique structures consist of Si atoms at the vertices of a three-dimensional cage, with O atoms bridging the silicon units.^29−31^ Their molecular formula can be described as [RSiO_1.5_]_*n*_, where R represents the organic substituents attached to the silicon atoms. This versatile architecture allows for a wide range of structural variations and functionalizations, leading to diverse properties and applications. SQs exhibit exceptional thermal stability and tunable properties, making them attractive for use in fields such as catalysis, nanomaterials, coatings, and energy storage. Furthermore, their ability to integrate organic and inorganic components provides a platform for tailoring properties and designing advanced materials with enhanced performance and multifunctionality.^29−39^ There are some reports of the conjunction of silsesquioxane with ferrocene in diverse combinations, e.g., to form amphiphilic systems, grafted copolymers with magnetic properties, or porous materials for dye/heavy metal absorption features.^9,10,25,26,28,40−48^ Some of these systems exhibit interesting electrochemical properties, influencing their application. On the contrary, the distinct types of dendrimer cores should be also mentioned, but there are significantly lower numbers of papers concerning silsesquioxane-based dendrimers using ferrocene moieties as terminal fragments.^25,26,28^

**Scheme 1:**
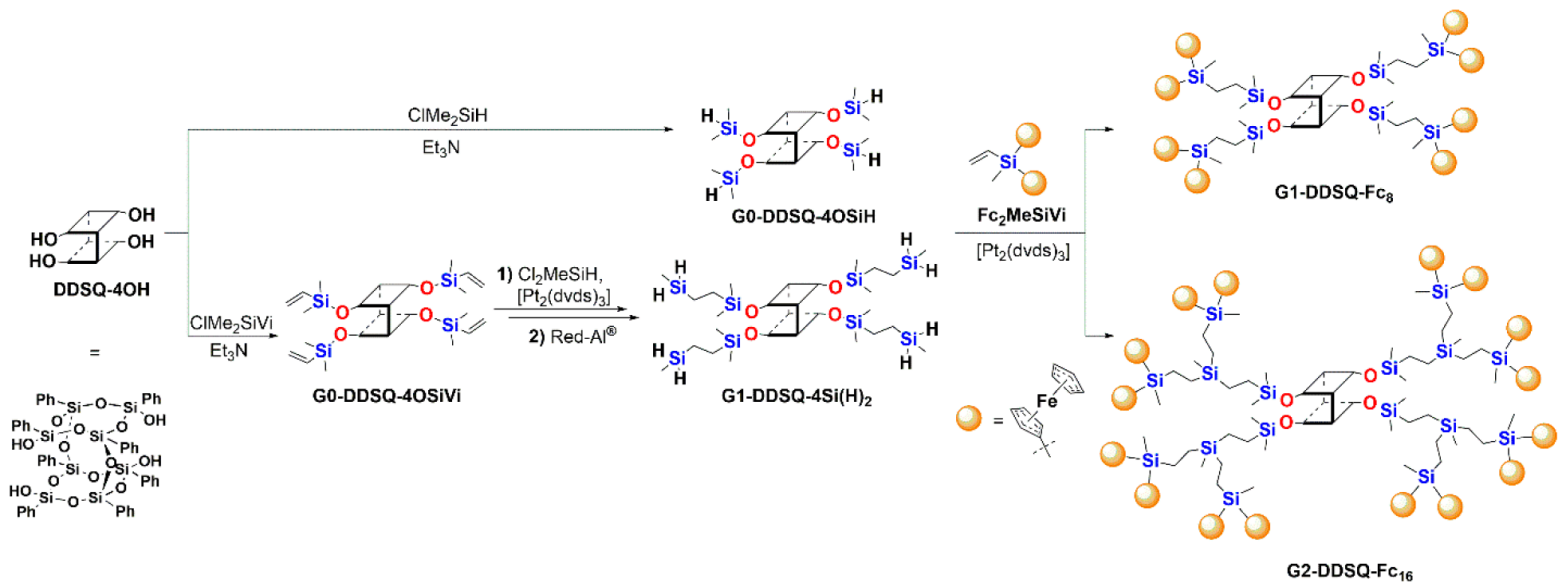
General Synthetic Route for Obtaining Ferrocene Dendrimers with Double-Decker Silsesquioxane Cores via Sequences of Reactions (condensation, reduction, and hydrosilylation)

Herein, we present studies on the synthesis of double-decker silsesquioxane molecules exhibiting electrochemical properties, i.e., possessing silylferrocene moieties. The synthetic protocol is based on a sequence of condensation, reduction, and hydrosilylation reactions (Scheme 1). The obtained compounds, varying in the amount of silylferrocene units attached to the inorganic Si–O–Si core, were analyzed in terms of their redox properties in solution as well as on Pt electrodes using cyclic voltammetry (CV) and electrochemical impedance spectroscopy (EIS) techniques.

## Results and Discussion

### Synthesis of Silylferrocene Dendrimers Anchored on a Double-Decker Silsesquioxane Core

The synthesis of DDSQ-based silylferrocene dendrimers was envisaged to exploit the DDSQ precursors equipped with four reactive arms terminated with either four (**G0-DDSQ-4OSiH**)^39^ or eight Si–H [**G1-DDSQ-4Si(H)**_**2**_]^32^ functionalities to be reacted with vinylsilyldiferrocene (**Fc**_**2**_**MeSiVi**).^49^ This enabled the highly efficient and selective formation of two silsesquioxane G1 and G2 dendrimers bearing either four or eight silylferrocene units attached to an open-cage DDSQ core.

The first generation of the ferrocene dendrimer (**G1-DDSQ-Fc**_**8**_) was synthesized with tetrafunctional DDSQ with four reactive Si–H groups **G0-DDSQ-4OSiH** and **Fc**_**2**_**MeSiVi** via hydrosilylation using the following stoichiometry and reaction conditions: [**G0-DDSQ-4OSiH**]:[**Fc**_**2**_**MeSiVi**]:[Pt_2_(dvds)_3_] = 1:4.6:6 × 10^–4^, 95 °C, 24 h. Note that the reactivity of the Si–vinyl moiety was influenced by the steric hindrance and electronic effects of the ferrocene units. It was manifested by the presence of a residual resonance line at 4.85 ppm, signifying unreacted Si–H moieties. To achieve full conversion of **G0-DDSQ-4OSiH**, a 20% excess of **Fc**_**2**_**MeSiVi** per Si–H group and a catalyst loading of 4 × 10^–3^ are necessary.

**Figure 1:**
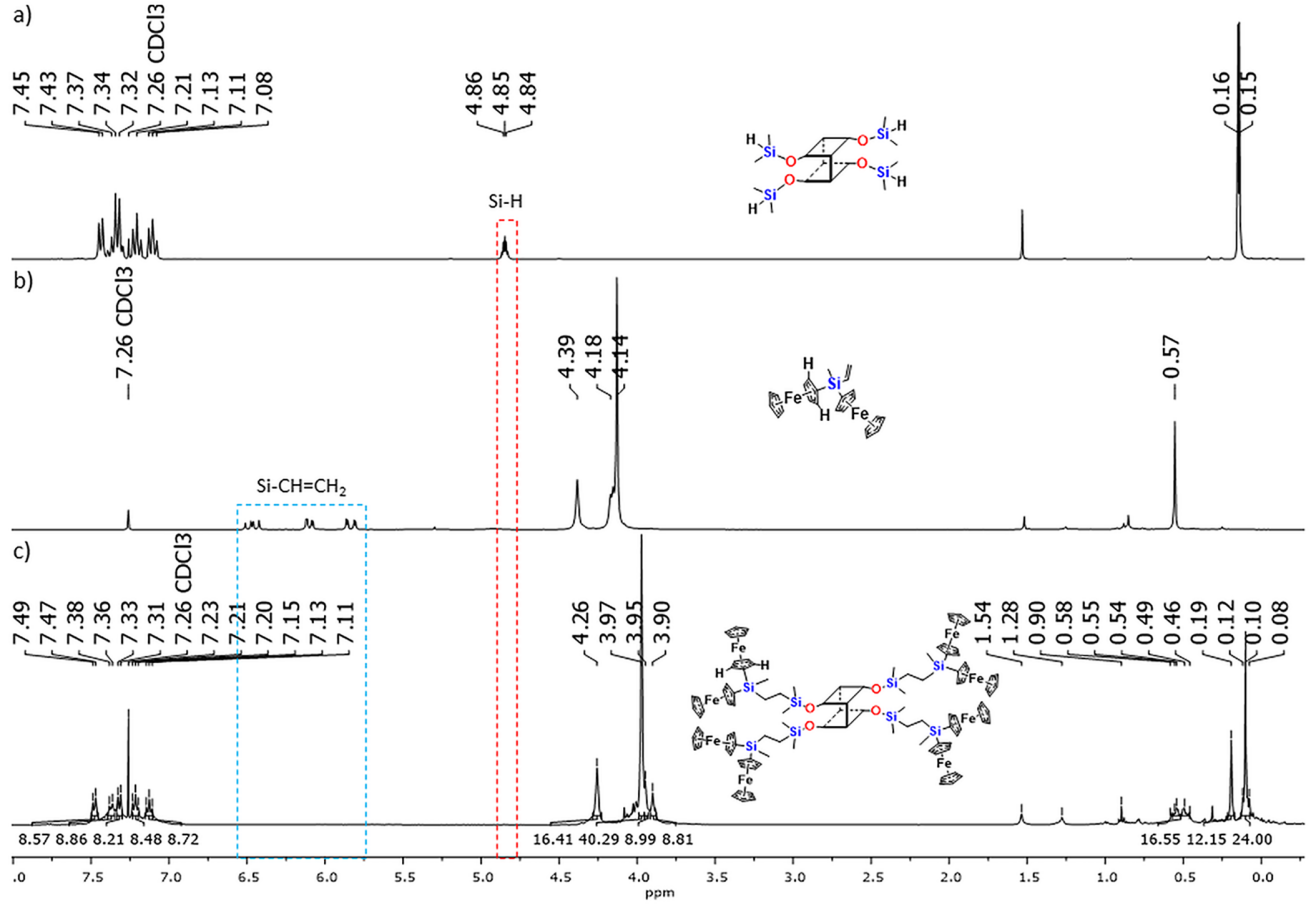
Selected range of stacked ^1^H NMR spectra of (a) **G0-DDSQ-4OSiH**, (b) **Fc**_**2**_**MeSiVi**, and (c) **G1-DDSQ-Fc**_**8**_.

The Fourier transform infrared (FT-IR) spectrum of the resulting mixture showed the disappearance of the Si–H bond in the area ca. υ̅ = 2100 cm^–1^ and υ̅ = 900 cm^–1^ confirming the complete conversion of **G0-DDSQ-4OSiH** (see Figure S1). A respective comparison of the ^1^H NMR spectra of **G0-DDSQ-4OSiH**, **Fc**_**2**_**MeSiVi**, and product **G1-DDSQ-Fc**_**8**_ is depicted in Figure 1. It confirms the disappearance of the resonance line from Si–H (4.85 ppm) and Si–CH=CH_2_ (5.84 ppm, *J*_HH_ = 20.3, 3.7 Hz; 6.10 ppm, *J*_HH_ = 14.6, 3.7 Hz; 6.47 ppm, *J*_HH_ = 20.3, 14.6 Hz) in product **G1-DDSQ-Fc**_**8**_ (Figure 1c). Interestingly, changes were observed in the ferrocene fragment range (3.80–4.40 ppm) in **G1-DDSQ-Fc**_**8**_. Surprisingly, a low-field shift of the C–H peak in the cyclopentadienyl ring connected to the Si atom was detected when compared to that of substrate **Fc**_**2**_**MeSiVi**. It was not observed for similar ferrocene-containing systems with cubic T_8_-type silsesquioxane-based carbosilane dendrons.^25^ A possible explanation for this could be the difference in the chemical surroundings of the silicon atoms (their order) attached to the SQ core. Also, the steric hindrance of the silyl–ferrocenyl groups is decreased due to the different DDSQ core. As a result, these aspects could influence the shielding effect.

Second-generation ferrocene dendrimer **G2-DDSQ-Fc**_**16**_ was synthesized in an analogous way. Silsesquioxane **G1-DDSQ-4Si(H)**_**2**_ was obtained in a one-pot reaction, i.e., hydrosilylation followed by reduction (Si–Cl to Si–H) (Scheme 1).^32^ The G2 dendrimer was synthesized with DDSQ bearing eight Si–H reactive groups using a 1:9.6:8 × 10^–3^ [**G1-DDSQ-4Si(H)**_**2**_]:[**Fc**_**2**_**MeSiVi**]:[Pt_2_(dvds)_3_] stoichiometry that enabled complete Si–H conversion. Respective stacked FT-IR and ^1^H NMR spectra of reagents confirm the obtained results (Figures S2 and S3).

**Figure 2:**
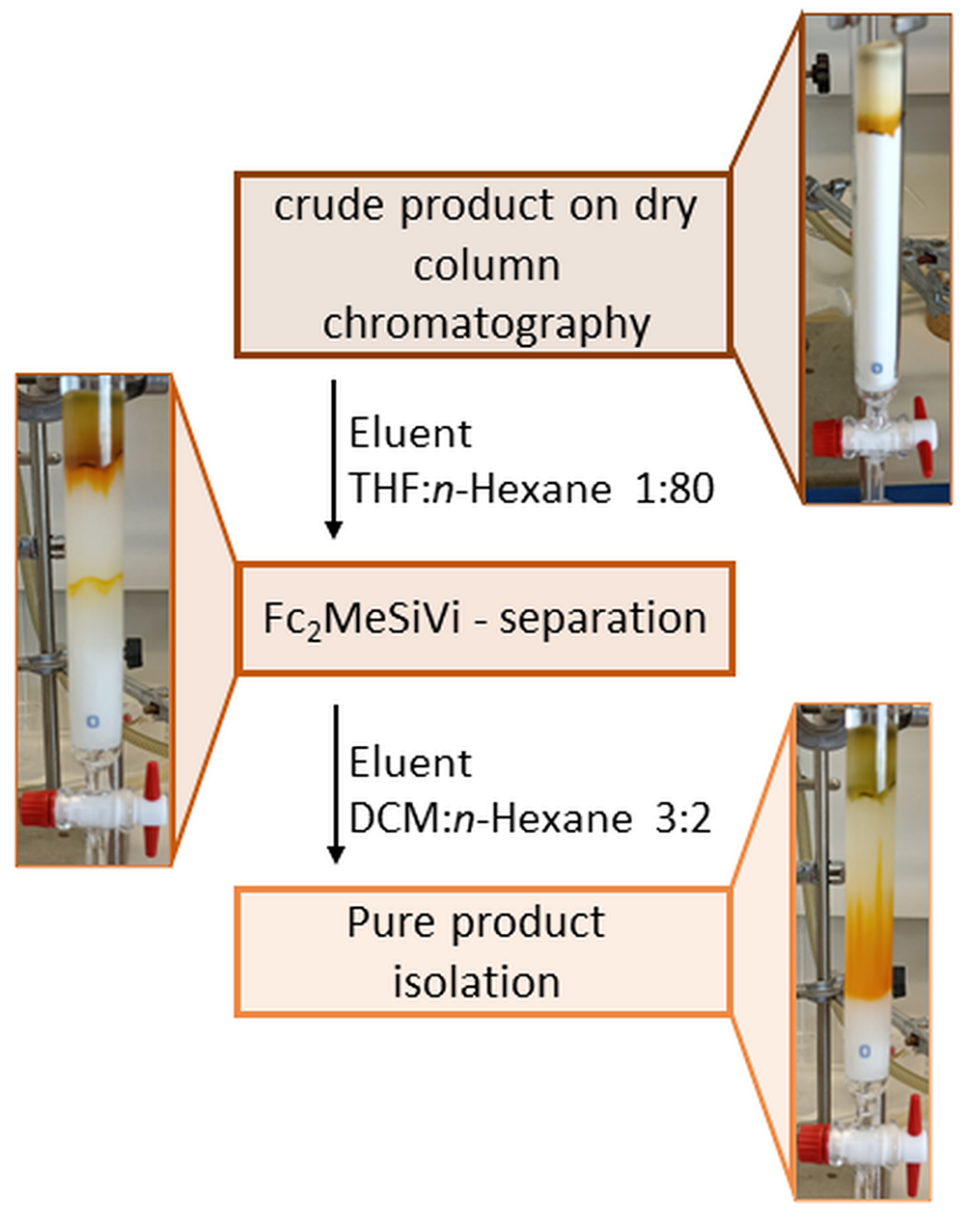
Schematic representation of the DDSQ-based ferrocene dendrimer purification procedure.

The most intriguing aspect of the synthesis of ferrocene dendrimers with a DDSQ core was the discovery of a suitable and efficient method for purifying the product from unreacted **Fc**_**2**_**MeSiVi**. Observable differences in solubility between product **G1-DDSQ-Fc**_**8**_ and substrate **Fc**_**2**_**MeSiVi** were noted in basic organic solvents. Substrate **Fc**_**2**_**MeSiVi** is soluble in common organic solvents, e.g., DCM, THF, toluene, or *n*-hexane. However, double-decker silsesquioxane **G1-DDSQ-Fc**_**8**_, characterized by an inorganic Si–O–Si core with exposed phenyl rings, does not dissolve in *n*-hexane, MeOH, or MeCN (Table S1). This fact enables us to elaborate an efficient isolation methodology using column chromatography. First, the reaction mixture was dissolved in a minimum volume of DCM and applied to a dry SiO_2_ column, and the solvent was allowed to evaporate overnight. The next day, the use of a THF/*n*-hexane eluent in a 1:80 ratio facilitated the efficient removal of **Fc**_**2**_**MeSiVi**, while product **G1-DDSQ-Fc**_**8**_ remained on the top of the chromatography column. The eluent was then changed to 3:2 DCM/*n*-hexane, and the product was easily eluted. Because both compounds are colorful, their separation was clearly visible, which is depicted in Figure 2. The product yields for **G1-DDSQ-Fc**_**8**_ and **G2-DDSQ-Fc**_**16**_ were quite high (74% and 60%, respectively). The structures of both products were confirmed by spectroscopic analyses as well as MALDI-TOF MS, which demonstrated the molecular ions at *m*/*z* 3062.3 and 5112.6, respectively (details in Figures S4–S11).

**Figure 3:**
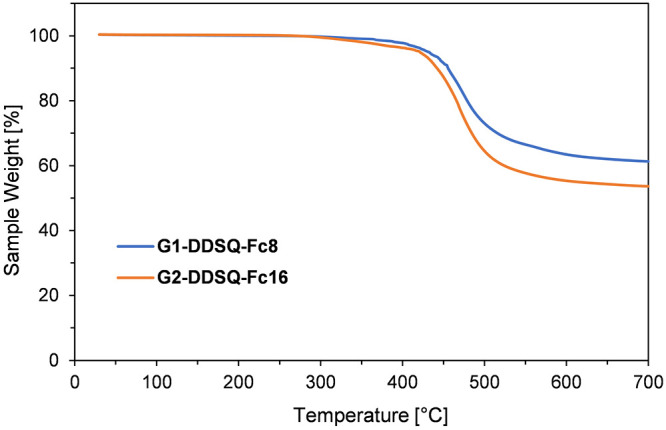
Thermogravimetric analysis of **G1-DDSQ-Fc**_**8**_ (blue) and **G2-DDSQ-Fc**_**16**_ (orange) performed in nitrogen.

Dendrimers **G1-DDSQ-Fc**_**8**_ and **G2-DDSQ-Fc**_**16**_ exhibited a high thermal stability. For **G1-DDSQ-Fc**_**8**_, only one degradation step was observed, starting at 326 °C, characterized by a *T*_d_^5%^ of 431 °C and a *T*_d_^10%^ of 455 °C. The major mass loss was detected in the 380–580 °C temperature range. However, for **G2-DDSQ-Fc**_**16**_, the mass loss began at 290 °C and *T*_d_^5%^ = 421 °C and *T*_d_^10%^ = 442 °C with the major mass loss in the range of 420–600 °C (Figure 3 and Table S2). This observation follows the literature for two analogous ferrocenyl carbosilane dendrimers but with an octasubstituted T_8_-type SQ core.^25^ It could be a higher-organic content derivative in the case of **G2-DDSQ-Fc**_**16**_ versus **G1-DDSQ-Fc**_**8**_. Moreover, at 700 °C the amount of residue was larger for **G1-DDSQ-Fc**_**8**_ (61%) than for **G2-DDSQ-Fc**_**16**_ (54%) due to the smaller amount of organic, aliphatic groups terminated with a ferrocene group. Interestingly, upon comparison of **G1-Fc**_**16**_,^25^ which possesses an octasubstituted T_8_-type core, and **G2-DDSQ-Fc**_**16**_, which features a double-decker T_12_-type core, both having the same number of Fc groups but different SQ cores, **G2-DDSQ-Fc**_**16**_ exhibited superior thermal stability. This observation might be attributed to the higher content of silicon atoms (due to the higher generation of the dendrimer) and the presence of phenyl groups at the DDSQ core, collectively contributing to the increased thermal stability of this system.^25,50^

### Electrochemical Study in Solution

Dendrimers containing redox-active moieties have been the subject of extensive research due to their intriguing properties and various applications, such as electron transfer mediators, catalysts and biocatalysts, molecular sensors, and electronic devices.^4,51^ Among them, ferrocene derivatives stand out for their notable characteristics, and their special ability to prepare modified electrodes, due to the change in their solubility associated with the ferrocene oxidation.^12^

**Figure 4:**
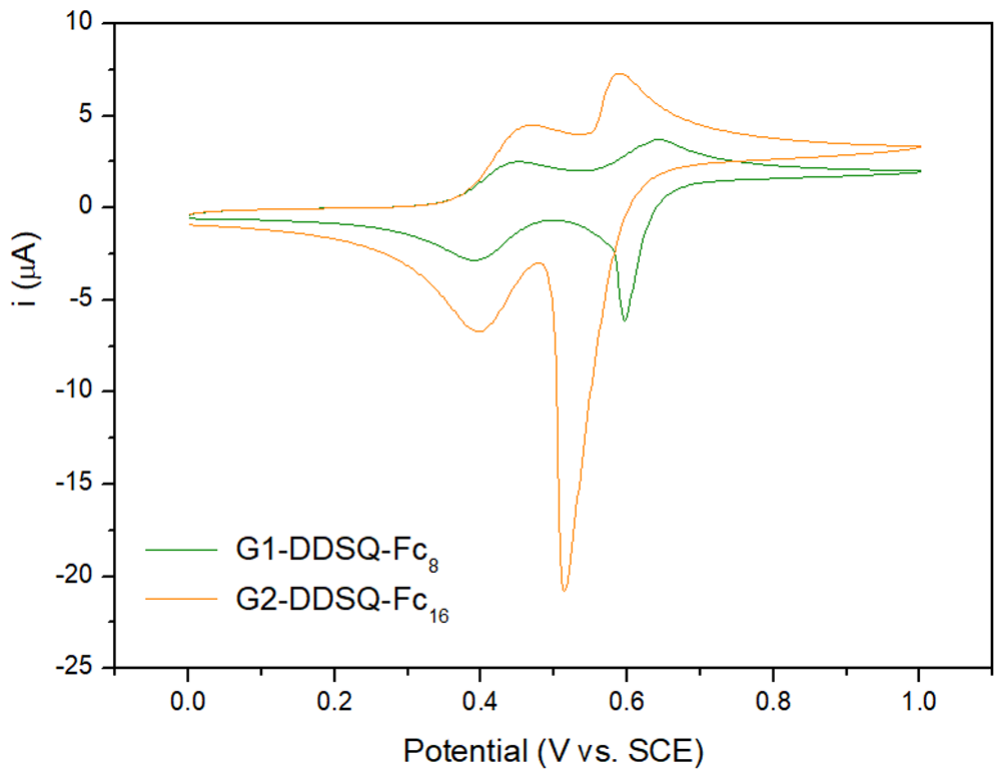
Cyclic voltammograms of dendrimers **G1-DDSQ-Fc**_**8**_ and **G2-DDSQ-Fc**_**16**_ in CH_2_Cl_2_ with a 0.1 M *n*-Bu_4_NPF_6_ solution (scan rate of 20 mV s^–1^).

To study the electrochemical properties, the new dendrimers were tested by using CV in DCM solution at a low concentration of ferrocene centers (∼10^–4^ M). The choice of DCM as the solvent was made on the basis of our extensive experience with other ferrocenyl dendrimers.^5,6,11,12,25^ These systems exhibit high solubility in DCM, and the wide electroactivity range of this solvent makes it highly suitable for investigating electrochemical systems involving interacting ferrocene groups. The cyclic voltammograms of both compounds (Figure 4) showed two well-separated and reversible oxidation peaks of equal intensity at *E*_1_^0^ = 0.42 V and *E*_2_^0^ = 0.62 V for **G1-DDSQ-Fc**_**8**_ and *E*_1_^0^ = 0.42 V and *E*_2_^0^ = 0.55 V for **G2-DDSQ-Fc**_**16**_ (vs the saturated calomel electrode). Both compounds show, upon reversal of the scan after the second oxidation process, a narrow reduction wave indicating the presence of a stripping peak. As expected, the oxidized form of DDSQ ferrocene derivatives precipitates on the surface of the electrode, and during the reverse scan, the film partially redissolves as they are reduced. This observation is also in good accord with the literature. The two-wave redox response and the change in solubility that accompanied the change in oxidation state were published for similar systems, e.g., oligo- and poly(ferrocenylsilanes) or polysiloxanes, block copolymer, and carbosilane dendrimers contain diferrocenylsilane units.^11,25,27,49,52,53^ As a consequence of this change in solubility, during continuous scanning, the peak currents increase, especially for dendrimer **G2-DDSQ-Fc**_**16**_, indicating the formation of a film on the electrode (Figures S12 and S13). It is worth noting that the behavior of **G1-DDSQ-Fc**_**8**_ and **G2-DDSQ-Fc**_**16**_ is different from that of a simple carbosilane-based dendrimer or a dendrimer with an octa-T_8_SQ core with 8 or 16 ferrocene groups, respectively.^11,25^

The plots of peak current versus the squared scan rate (ν^1/2^) were linear in all cases (see Figures S14 and S15), which are indicative of diffusion-controlled redox processes. The diffusion coefficients, *D*_0_, for both systems were calculated by means of the Randles–Sevick equation (eq 1)
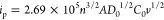
1were *D*_0_ = 1.44 × 10^–4^ cm^2^ s^–1^ and *D*_0_ = 4.41 × 10^–4^ cm^2^ s^–1^ for **G1-DDSQ-Fc**_**8**_ and **G2-DDSQ-Fc**_**16**_, respectively. This difference can explain the lesser growth of the **G1-DDSQ-Fc**_**8**_ current in successive cycles shown in Figures S12 and S13.

On the contrary, the two-wave redox response confirms the existence of interactions between two ferrocenyl centers connected by the bridging silicon atom. The first oxidation occurs in nonadjacent sites, which prevents the oxidation of the remaining ferrocene centers adjacent to the already oxidized ones. To determine the degree of interaction between the two iron sites, the redox potential difference (Δ*E*_2–1_^o^ = *E*_2_^o^ – *E*_1_^o^) was calculated and equals 200 mV for **G1-DDSQ-Fc**_**8**_ and 130 mV for **G2-DDSQ-Fc**_**16**_. Moreover, the partially oxidized silsesquioxanes can be classified as class II mixed-valence species according to the Robin–Day classification, and comproportionation constant *K*_c_ equals 2405 mV for **G1-DDSQ-Fc**_**8**_ and 158 mV for **G2-DDSQ-Fc**_**16**_, corresponding to the equilibrium^54−56^
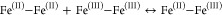


### Electrochemical Studies of Modified Electrodes

**Figure 5:**
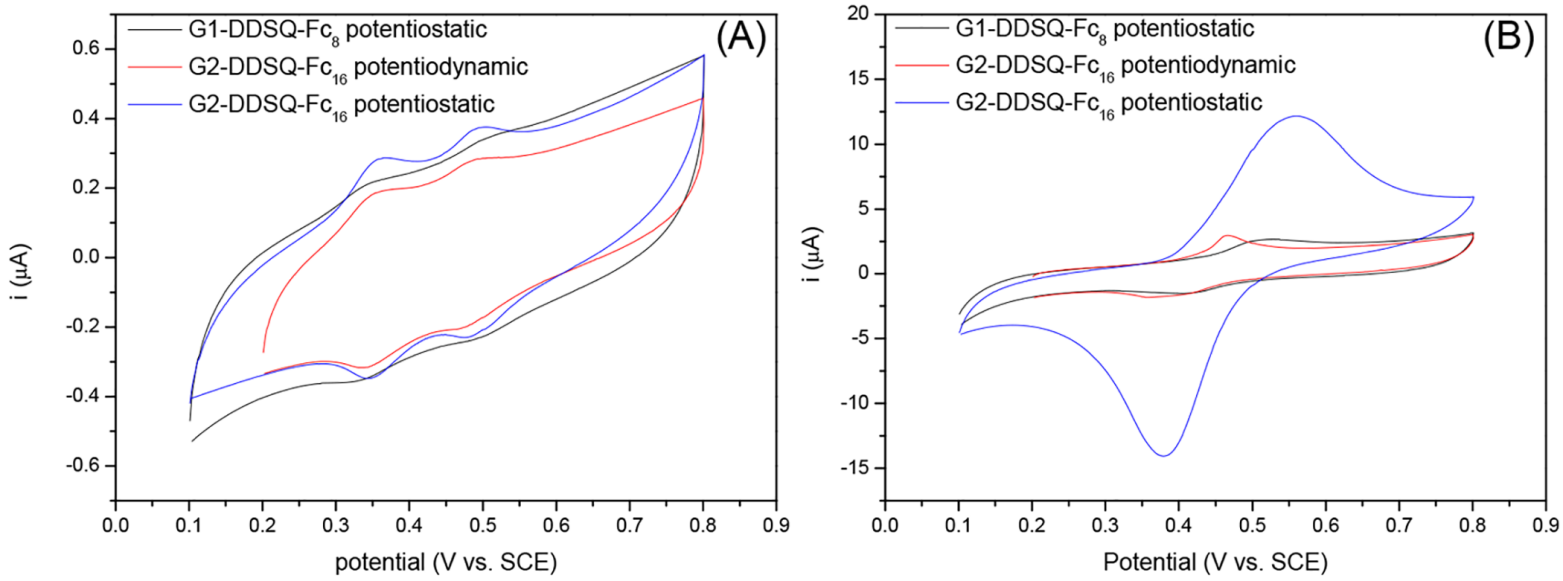
Steady-state voltammetric response of electrodes modified with both dendrimers (A) in CH_2_Cl_2_ with a 0.1 M *n*-Bu_4_NPF_6_ solution and (B) in phosphate buffer (pH 7.0) and a 0.1 M NaClO_4_ solution (scan rate of 20 mV s^–1^).

As cited above, an interesting aspect of dendrimers functionalized with ferrocene is the possibility of depositing them on several electrode surfaces to obtain electroactive films. Because both dendrimers have the same core-type structure and their electroactivity is solely attributed to the presence of ferrocene groups, it is anticipated that any variations in the electrochemical properties of both modified electrodes will depend primarily on the ferrocene:dendrimer ratio (active centers per film volume) and the characteristics of the films formed. These factors may collectively influence the electron transfer through the film, that is, potentially leading to differences in the electrochemical kinetics of the modified electrodes. The amount of electrodeposited material depends on the amount of electricity consumed in the electrolytic deposition (Faraday’s law), and this in turn can be controlled by the applied potential, the application time, and the concentration of the electroactive substance in the bulk solution. On the contrary, the structure of the obtained film can be different depending on the mode of application of the potential. When the diffusion of the dendrimer is fast enough, the potentiodynamic method (potential sweeps) allows more permeable films to be obtained, because it facilitates the formation of the polymer at nucleation sites in an orderly manner. This procedure allows precise control of the film thickness based on the number of scans. However, if the diffusion coefficient is too slow and/or the proportion of ferrocene groups is low in relation to the size of the molecule, it can be necessary to use a potentiostatic (constant potential) method with a sufficient overpotential to counteract a low level of diffusion. In this case, the films obtained are usually more compact, and the control of their thickness, based on the time, should be more difficult. For this reason, we tried to prepare electrodes by both procedures. Initially, to modify the first electrode with **G1-DDSQ-Fc**_**8**_, the potentiodynamic method was employed. However, the film thickness obtained was too small (Γ = 1.98 × 10^–12^ mol of Fc/cm^2^). Consequently, controlled-potential electrolysis was investigated, and an applied potential of 1.0 V for 5 min was chosen as the most appropriate condition. In the case of **G2-DDSQ-Fc**_**16**_, both methods can be used, through repeat cycling (20 cycles between 0.0 and 1.0 V), and controlled-potential electrolysis at 1.0 V for 5 min was the best condition. Figure 5 shows the cyclic voltammograms of the electrodes modified with both dendrimers by the described procedures.

As one can see, in a non-aqueous solution, the cyclic voltammograms of all of the obtained modified electrodes also presented the two successive well-defined reversible oxidation–reduction systems, which confirm the existence of interactions between the two iron centers. In addition, one can see that the sharp peak, indicating partial redissolution due to the reduction of ferrocenium, no longer appears. This reveals the electrochemical stability of the films. Effectively, as has already been observed with other ferrocenyl dendrimers sharing similar structures and/or compositions,^5,6,11,12,25^ the films of these systems remain stable even in DCM. They attain their steady-state cyclic voltammograms from the first cycle, and this stability is maintained in successive cycles without any mass loss. The formal potential values, *E*_1_^0^ and *E*_2_^0^, calculated as the average of anodic and cathodic peak potentials, for all modified electrodes at a slow scan rate (10 mV s^–1^), are listed in Table 1. Note that the **G2-DDSQ-Fc**_**16**_ films show more defined ferrocenyl peaks due to the higher proportion of ferrocene groups in the molecule.

**Table 1:** Electrochemical Data of Modified Electrodes

	**G1-DDSQ-Fc**_**8**_		**G2-DDSQ-Fc**_**16**_			
	potentiostatic		potentiodynamic		potentiostatic	
	aqueous	non-aqueous	aqueous	non-aqueous	aqueous	non-aqueous
*E*_pa1_ (V)	0.48	0.36	0.45	0.35	0.51	0.36
*E*_pc1_ (V)	0.42	0.33	0.37	0.34	0.39	0.34
*ΔE*_p1_ (mV)	65	30	80	10	120	20
*E*_1_^0^ (V)	0.45	0.34	0.41	0.35	0.45	0.35
*E*_pa2_ (V)	–	0.51	–	0.49	–	0.49
*E*_pc2_ (V)	–	0.50	–	0.48	–	0.47
*ΔE*_p2_ (mV)	–	10	–	10	–	20
*E*_2_^0^ (V)	–	0.50	–	0.49	–	0.48
α	0.47	0.51	0.51	0.52	0.54	0.51
*n*	0.98	1.06	0.93	1.10	1.07	1.23
*k*_s_ (s^–1^)	1.02	–	2.03	–	0.09	–

In aqueous solution, the obtained voltammograms with the same modified electrodes showed only one reversible system, which demonstrates the overlapping interaction between the ferrocenyl units (Figure 5B). The presence of polar solvent molecules, which can be situated between the ferrocene groups, counteracts the electrochemical interactions, inhibiting the connection of two redox couples. Consequently, only one system appears. A similar observation was reported for poly(dialkylsilylenefferocenylene), siloxane homopolymers, block copolymer films, or other ferrocene dendrimers.^5,6,11,25,52,53^ The obtained formal potentials are also listed in Table 1.

As the electrodes used in panels A and B of Figure 5 are the same, it is clear that the electroactivity of the films improves remarkably in aqueous media. For this reason, the cyclic voltammograms in an aqueous medium will be used to calculate the coatings of the films. The film thicknesses obtained were around 3 × 10^–10^ mol of Fc/cm^2^ for dendrimer **G1-DDSQ-Fc**_**8**_ (potentiostatic method) and 8 × 10^–10^ and 7 × 10^–9^ mol of Fc/cm^2^ for dendrimer **G2-DDSQ-Fc**_**16**_ (potentiodynamic and potentiostatic methods, respectively).

On the contrary, it is also possible to observe in Figure 5B the difference between the films of **G2-DDSQ-Fc**_**16**_ obtained by the different procedures. This observation suggests that the thicker film obtained by the potentiostatic method facilitates counterion movement and electron transfer without a concomitant resistance increment. That is, we can predict that the transfer of electrons is very fast, despite the thickness of the film. Finally, we confirm the difficulty of deposition of dendrimer **G1-DDSQ-Fc**_**8**_, due to the lower Fc:molecule ratio, despite using a potentiostatic method.

To study the kinetics of electron transfer through the films and characterize the redox systems in both media and in relation with preparation methods, the number of electrons exchanged and the electronic transfer coefficients, α, indicative of symmetry of the electrochemical systems, were estimated from the peak width at midheight, *W*_1/2_. At very low scan rates (10 mV s^–1^), the cathodic and anodic peaks should approach the reversible peaks, symmetrical with respect to the potential axis. The midheight, denoted as *W*_1/2_, becomes independent of α and approaches 90.6/*n* mV. At high scan rates (500 mV s^–1^), the cathodic peak midheight must be equal to 62.5/*n*α, whereas the anodic peak midheight must be equal to 62.5/*n*(1 – α).^57^ All systems show the exchange of one electron, corresponding to the ferrocene/ferrocenium redox system, and α coefficients close to 0.5, corresponding to symmetric and reversible systems. The average obtained values are listed in Table 1.

Usually, the kinetics of modified electrodes with mono- or multilayers strongly adsorbed on an electrode surface can be studied by means of the model developed by Laviron.^57,58^ The model is based on the variation of the cyclic voltammograms with the scan rate (*v*), and let us estimate if the electroactive groups are surface confined. If this occurs, the peak currents must show a linear relationship with *v* in some scan rate intervals.^59^ In addition, the linearity at high rates proves the exhaustive oxidation–reduction through the film.^60^ On the contrary, the study of the variation of the peak potentials with *v* provides significant information about the kinetics of the modified electrodes. The variation (or not) of the peak potentials versus *v* allows us to evaluate the existence of kinetic limitations and, where appropriate, to estimate homogeneous standard rate constant *k*_s_ for the electron transfer redox system.

**Figure 6:**
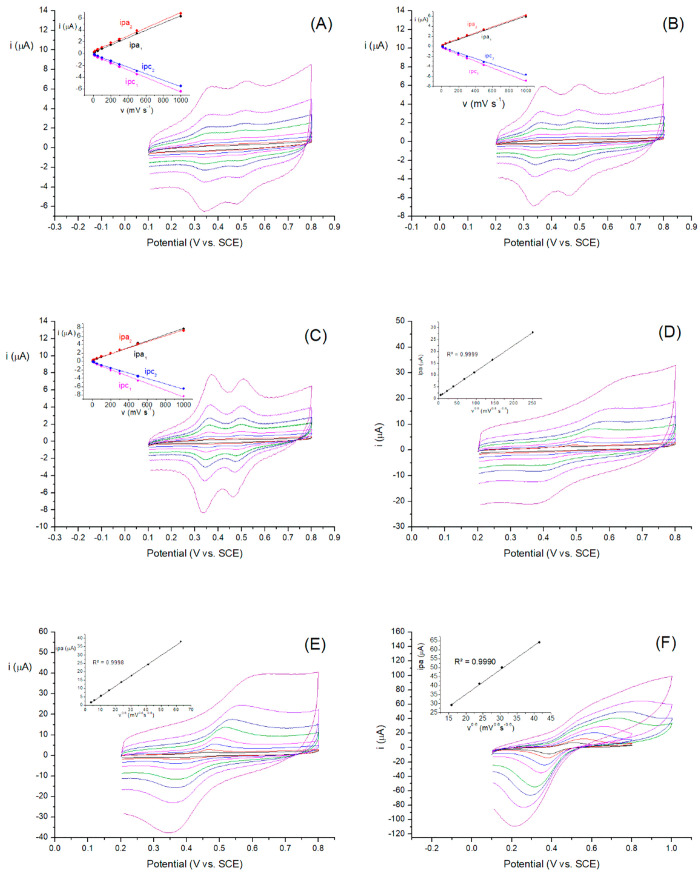
Dependence of the peak current on the scan rate of (A) **G1-DDSQ-Fc**_**8**_ potentiostatic, (B) **G2-DDSQ-Fc**_**16**_ potentiodynamic, and (C) **G2-DDSQ-Fc**_**16**_ potentiostatic, modified Pt electrodes in CH_2_Cl_2_ with 0.1 M *n*-Bu_4_NPF_6_. Insets show the linear dependence of the peak currents on the scan rate. (D) **G1-DDSQ-Fc**_**8**_ potentiostatic, (E) **G2-DDSQ-Fc**_**16**_ potentiodynamic, and (F) **G2-DDSQ-Fc**_**16**_ potentiostatic, modified Pt electrodes in phosphate buffer (pH 7.0) and 0.1 M NaClO_4_. Insets show the linear dependence of the peak current on *v*^*x*^. Scan rates of 10, 20, 50, 100, 200, 300, 500, and 1000 mV s^–1^.

Figure 6 shows the cyclic voltammograms obtained in aqueous and non-aqueous media with all of the modified electrodes. The figures show very different behaviors in each medium. As one can see, all electrodes in a non-aqueous solution (Figure 6A–C) show cathodic and anodic peak separations (Δ*E*_p_) that are practically constant as the scan rate increases, and the Δ*E*_p_ values are only marginally greater than zero (Table 1). This fact indicates the absence of kinetic limitations.^61,62^ In addition, a linear relationship between the peak currents and the scan rate was observed (insets in the graphs of Figure 6), indicating the surface-confined nature of the ferrocenyl groups and the nonexistence of charge percolation. In other words, the films show behavior like that of a monolayer film.^28^

In aqueous media (Figure 6D–F), the peak potentials shift with an increase in scan rate. The study of this variation allows us to estimate *k*_s_. For this study, Laviron distinguishes two cases as a function of the magnitude of Δ*E*_p_.^57^ For the systems, and *v* values, for which *ΔE*_p_ > 200/*n* mV, *k*_s_ is calculated by eq 2:
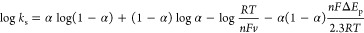
2where *R* is the gas constant, *F* is the Faraday constant, and *T* is the absolute temperature. For systems, and *v* values, for which *ΔE*_p_ < 200/*n* mV, Laviron provides a table with values of Δ*E*_p_ as a function of parameter *m*^–1^, where *m* is equal to *RTk*_s_/*Fnv*. The polynomial fit (*R*^2^ = 0.9992) of these data is calculated from eq 3:
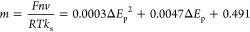
3In our case, the **G1-DDSQ-Fc**_**8**_ potentiostatic and the **G2-DDSQ-Fc**_**16**_ potentiodynamic films show *ΔE*_p_ values of <200 mV in the 10–300 mV s^–1^ interval, while the **G2-DDSQ-Fc**_**16**_ potentiostatic film Δ*E*_p_ was >200 for scan rates of >50 mV s^–1^. The obtained homogeneous rate constants are listed in Table 1. From these results, we can deduce that, in aqueous media, the **G2-DDSQ-Fc**_**16**_-modified electrode created by potentiodynamic method showed the best behavior.

With regard to the dependence of the peak current on the scan rate, Laviron demonstrated that, with multilayer films, the anodic and cathodic peak currents show a linear tendency for only large or small *v*. However, there is a middle range where *i*_p_ becomes proportional to *v*^*x*^, taking *x* values between 0.6, a value predicted by Laviron for multilayer films with fast electron transfer, and the unit (nonexistence of charge percolation in monolayer, or assimilated, films). As shown in the insets of Figure 6D–F, the studied systems present a good linear relation between the anodic or cathodic peak (not shown) currents on *v*^0.6^ over the whole sweep rate interval for the **G2-DDSQ-Fc**_**16**_ electrodes prepared by both methods, and on *v*^0.8^ for the **G1-DDSQ-Fc**_**8**_-modified electrodes. Consequently, in agreement with Laviron’s model, we can assert that the modified electrodes have a multilayer structure with fast electron transfer.

### Electrochemical Impedance Spectroscopy (EIS)

Another useful electrochemical method for measuring the interfacial properties present on the electrode surface is electrochemical impedance spectroscopy (EIS). This technique allows us to model the electrode–electrolyte interface using an equivalent circuit composed of the charge transfer resistance, which controls the electron transfer kinetics, *R*_CT_, the Warburg impedance, which represents the diffusion of ions from the bulk electrolyte to the electrode interface, *W*, the interfacial capacitance of the double layer, *C*_dl_, and the electrolyte resistance, *R*_s_.^63^

The Nyquist plots show the imaginary versus the real part of the impedance, which is used widely to estimate *R*_CT_ and *C*_dl_. The plot consists of a semicircular part, whose diameter represents *R*_CT_, and a linear part at low frequencies, characteristic of diffusion-controlled systems. Nonetheless, for rough surfaces, *C*_dl_ cannot describe the electronic properties of the interface correctly, because the system deviates from the ideal capacitive behavior. In these cases, one must introduce a constant phase element, CPE, that reflects the nonhomogeneity of the layer and is defined as CPE = *A*^–1^(*jw*)^*–n*^, where *n* is the interface deviation from the Randles model, taking values between 0.5 and 1, and *A* is a coefficient that becomes equal to *C*_dl_ when *n* = 1.

**Figure 7:**
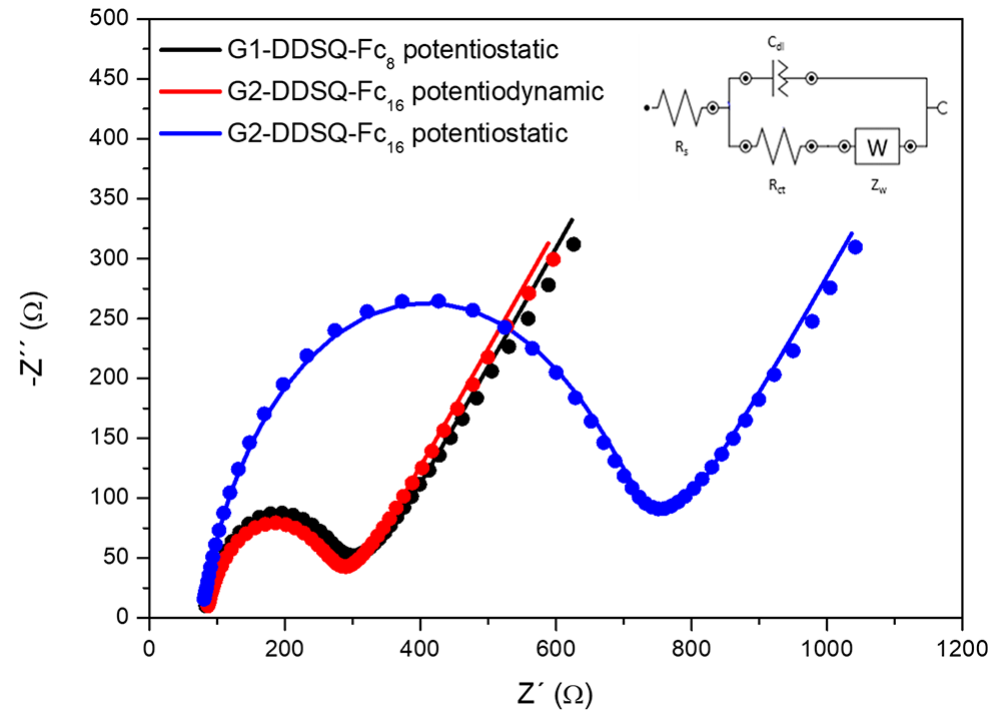
Nyquist plot of modified electrodes in 0.1 M KCl with a 10 mM K_3_[Fe(CN)_6_]/K_4_[Fe(CN)_6_] solution. Lines are the fits and simulations to obtain the Randles-type equivalent circuits. The inset shows a model of an equivalent circuit obtained from fit and simulation of the EIS data.

From the Nyquist plots, we confirm the previous conclusions about the modified electrodes with both dendrimers by both procedures. The best film, in relation to electron transfer kinetics, was the potentiodynamic **G2-DDSQ-Fc**_**16**_-modified electrode, with a smaller *R*_CT_, while the same material prepared by the potentiostatic method showed the highest value (Figure 7). Table 2 collects all of the EIS results for the three types of electrodes, in comparison with those corresponding to a Pt bare electrode.^7^ The equivalent circuits for each electrode are shown in Figure S16.

**Table 2:** EIS Results for the Three Types of Modified Electrodes

electrode	*R*_ct_ (Ω)	CPE (μF)	*n*	*i*_0_ (μA)	*k*^0^ (cm s^–1^)	χ^2^
bare Pt^7^	32	4.29	0.829	791	1.17 × 10^–2^	–
potentiostatic **G1-DDSQ-Fc_8_**	214	11.00	0.834	132	2.34 × 10^–6^	0.0095
potentiodynamic **G2-DDSQ-Fc**_**16**_	194	9.48	0.843	144	2.53 × 10^–6^	0.0025
potentiostatic **G2-DDSQ-Fc**_**16**_	643	5.40	0.862	42.4	7.29 × 10^–7^	0.0056

From the EIS data, we can calculate other important parameters such as the exchange currents (*i*_0_) and electron transfer heterogeneous constants (*k*^0^). The first parameter is calculated from eq 4:
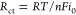
4where *R* is the gas constant, *T* is the absolute temperature, *F* is Faraday’s constant, and *k*^0^ is then obtained from eq 5:
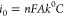
5where *C* stands for the concentration of the electroactive species in moles per cubic centimeter and *A* the electrode area in square centimeters.^64^

The obtained values (Table 2) again demonstrate that the best conductive and kinetic properties are obtained with the **G2-DDSQ-Fc**_**16**_ dendrimer electrodeposited by the potentiodynamic method.

### Scanning Electron Microscopy (SEM) Analysis

**Figure 8:**
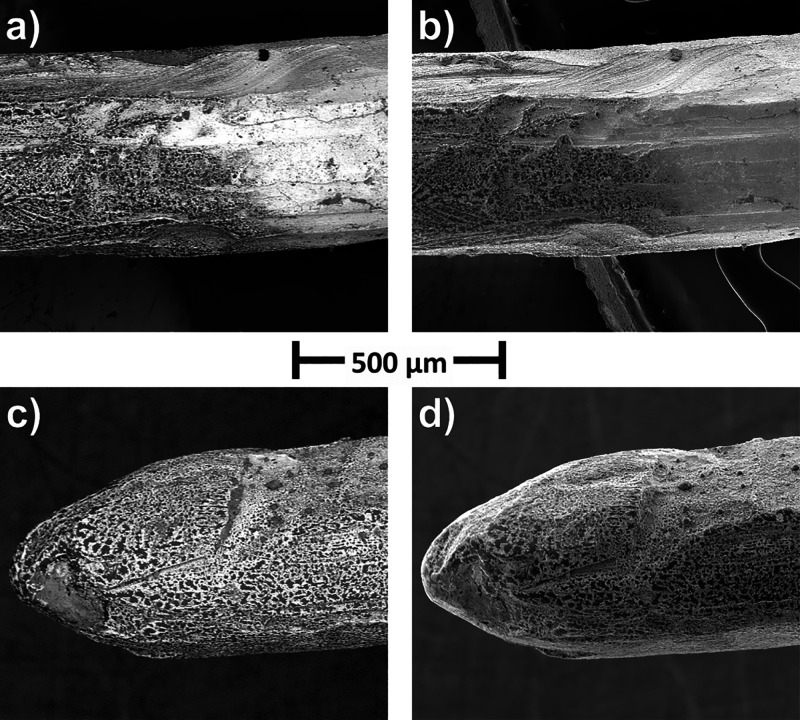
Scanning electron microscopy images of Pt wires modified by **G1-DDSQ-Fc**_**8**_ using a controlled potential method at an *E* of 1.0 V for 5 min: (a and c) BSE (back-scattered electrons) images and (b and d) SE (secondary electron) detector.

The morphology of Pt electrodes modified with **G1-DDSQ-Fc**_**8**_ and **G2-DDSQ-Fc**_**16**_ was studied using scanning electron microscopy (SEM) (Figure 8 and Figure S15). The electrodes were prepared by controlled-potential electrolysis (*E* = 1.0 V for 5 min) and repeat cycling (20 potential cycles between 0.0 and 1.0 V). Both methods, cyclic voltammetry and electrochemical impedance spectroscopy, demonstrated the formation of multilayer films on the electrodes. The SEM images show how the films completely cover the surface of the platinum with an appreciable thickness compatible with a multilayer coating. Figure 8 and Figure S17a,b display the part of the Pt wire of which the right side was unimmersed in the ferrocene dendrimer solution and the left side of which the film was formed.

**Figure 9:**
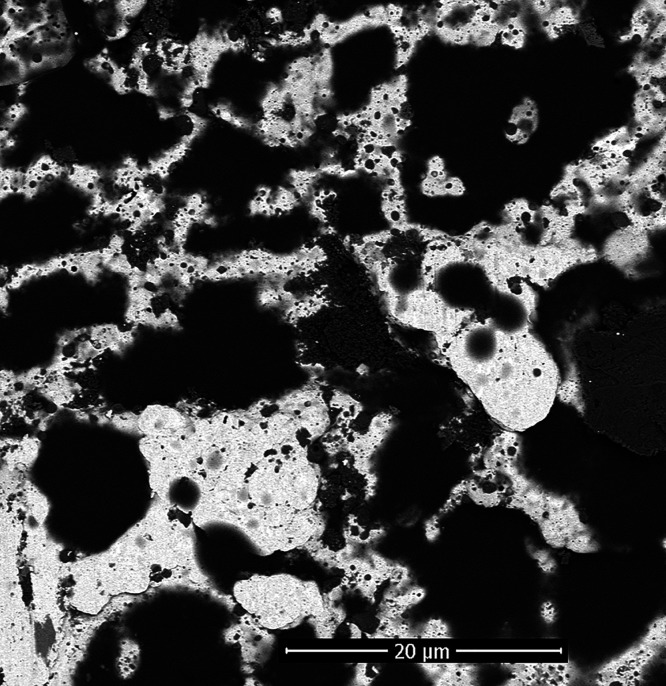
Magnification of scanning electron microscopy BSE detection images of a Pt wire modified by **G1-DDSQ-Fc**_**8**_.

The brightness of the pixels in a BSE image is closely related to the atomic mass of the nuclei of which the area is composed. This makes it possible to clearly distinguish between areas of pure platinum (bright) and areas of platinum covered by an organic layer. The layer covering the electrode is not smooth and uniform and shows an irregular granular surface with pores with cross-sectional sizes ranging from a few micrometers to 20 μm (Figure 9).

## Conclusions

In conclusion, this study successfully demonstrated the synthesis of novel metallodendrimers with double-decker silsesquioxane cores. The G1 and G2 silylferrocene derivatives were synthesized through a series of condensation, reduction, and hydrosilylation reactions. Spectroscopic (^1^H, ^13^C, and ^29^Si NMR and FT-IR) and spectrometric (MALDI-TOF MS) techniques confirmed the structures of the synthesized compounds, while their thermal stability and solubility in common organic solvents were also verified. Electrochemical characterization using cyclic voltammetry revealed two distinct, well-separated reversible redox processes in the non-aqueous solution, indicating the electrochemical activity of the metal sites within the dendrimers (**G1-DDSQ-Fc**_**8**_ and **G2-DDSQ-Fc**_**16**_).

Moreover, the modification of platinum electrodes was successfully achieved by employing either controlled-potential electrolysis or repeated cycling within a specific potential range. CV, EIS, and SEM imaging confirmed the formation of electroactive films of the utilized dendrimers on the platinum electrodes. It is worth noting that this study represents the first report on modifying a double-decker silsesquioxane with ferrocene groups and a dendrimer having such core and terminal groups.

In summary, this research significantly contributes to the understanding of the synthesis, characterization, and electrochemical properties of dendrimers with double-decker silsesquioxane cores, opening up avenues for their potential applications in various fields.

## Supporting Information Available

Materials, measurements, synthetic procedure, characterization data of the obtained products (Figures S4–S11), additional information about solubility (Table S1), thermogravimetric analysis (Table S2), cyclic voltammetry (Figures S12–S15), comproportionation constant (*K*_c_) calculations (eqs S1–S8), equivalent circuits (Figure S16), and SEM images (Figure S17) (PDF)
