# Supplementary material for: Metallodendrimers Unveiled: Investigating the Formation and Features of Double-Decker Silsesquioxane-Based Silylferrocene Dendrimers
Source: Inorg Chem. 2023 Sep 29;62(41):16932–42. doi: 10.1021/acs.inorgchem.3c02628 (PMC10583206; doi:10.1021/acs.inorgchem.3c02628)
Supplement: Supplementary file 1 — ic3c02628_si_001.pdf [file ic3c02628_si_001.pdf]

Supporting Information for

Metallodendrimers Unveiled: Investigating the Formation and Features of Double-Decker Silsesquioxane-Based Silylferrocene Dendrimers

Aleksandra Mrzygłód[a,b], M. Pilar García-Armada[c], Monika Rzonsowska[a,b], , Beata Dudziec*[a,b], Marek Nowicki[b,d]

[a]*Faculty of Chemistry, Adam Mickiewicz University in Poznan, Uniwersytetu Poznańskiego 8, 61-614 Poznan, Poland. E-mail:* beata.dudziec@gmail.com

[b]*Centre for Advanced Technologies, Adam Mickiewicz University in Poznan, Uniwersytetu Poznańskiego 10, 61-614 Poznan, Poland*

[c]*Departamento de Ingeniería Química Industrial, Escuela Técnica Superior de Ingenieros Industriales, Universidad Politécnica de Madrid, José Gutierrez Abascal 2, 28006 Madrid, Spain*

[d]*Institute of Physics, Poznan University of Technology, Piotrowo 3, 60-965 Poznan, Poland.*

Table of Contents:

1. General considerations S-2-
2. Measurements S-2-
3. Synthetic procedures S-4-
4. Additional spectra S-5-
5. Table of isolated compounds S-7-
6. Characterization data of the obtained products products (^{1}H, ^{13}C, ^{29}Si NMR spectra and IR spectroscopy) S-8-
7. Solubility of obtained products S-14-
8. TGA analysis S-14-
9. Cyclic Voltammetry studies S-15-
10. SEM images S-18-
11. References S-18-

1. General Considerations

Tetrasilanol form of DDSQ ($C_{48}H_{44}O_{14}Si_8$) (DDSQ-4OH), was purchased from Hybrid Plastics. Acetonitrile, dichloromethane (DCM), tetrahydrofuran (THF), methanol, toluene, chloroform-*d*, *n*-hexane, Karstedt's catalyst – 2% xylene solution, chlorosilanes (dichloromethylsilane, dichloromethylvinylsilane, chlorodimethylvinylsilane), 4 Å molecular sieves, triethylamine, acetic acid, Red-Al® and silica gel 60 were obtained from Sigma-Aldrich. Chlorodimethylsilanesilane was purchased from TCI. Ferrocene and *tert*-buthyllithium was obtained from Thermo Scientific. The following compounds were prepared according to the literature procedures: Fc_2MeSiVi, DDSQ-4OSiH, DDSQ-4OSiVi, DDSQ-4Si(H)$_2$[1–3]. All solvents were dried by calcium hydride (CaH_2) prior to use and stored under argon over 4Å molecular sieves. All liquid substrates were dried and degassed by bulb-to-bulb distillation. All reactions were carried out under argon atmosphere using standard Schlenk-line and vacuum techniques.

2. Measurements

Nuclear Magnetic Resonance (NMR)

^{1}H, ^{13}C, and ^{29}Si Nuclear Magnetic Resonance (NMR) were performed on Brucker Ultra Shield 400 and 300 spectrometers using $CDCl_3$ as a solvent. Chemical shifts are reported in ppm with reference to the residual solvents peaks for ^{1}H and ^{13}C and to TMS for ^{29}Si NMR.

FT-IR spectroscopy

Fourier Transform-Infrared (FT-IR) spectra were recorded on a Nicolet iS5 (Thermo Scientific) spectrophotometer equipped with a single reflection diamond ATR unit. In all cases, 16 scans at a resolution of 2 cm^{-1} were collected, to record the spectra in a range of 4000-650cm^{-1}.

MALDI-TOF MS

Matrix assisted laser desorption ionization time of flight (MALDI-TOF) mass spectrometry was performed using a Ultraflex TOF/TOF (Bruker Daltonics, Germany) in reflection mode. The thin-layer preparation method was applied. The matrix (2,5-dihydroxybenzoic acid - DHB) was dissolved at a concentration of 20 mg/mL in mixture of 0.1% TFA in de-ionized water (70 % v/v) and acetonitrile (30% v/v). The matrix solution was spotted onto the target and dried in air. In the next step sample solution (2 mg/mL in DCM) was deposited onto the matrix spot and dried in air.

Elemental analyses (EA)

Elemental analyses (EA) were performed using a Vario EL III instrument (Elementar Analysensysteme GmbH, Langenselbold, Germany).

Scanning electron microscopy (SEM)

Scanning electron microscopy (SEM) images of Pt -electrodes were taken using an FEI Quanta 250 FEG microscope. Images were taken in high vacuum mode, and accelerating voltage (5 kV). BackScattered Electrons (BSE) and Seconadary Electrons (SE) images have been taken.

Cyclic voltammetry (CV)

All the electrochemical measurements were performed using an Ecochemie BV Autolab PGSTAT 12, in dichloromethane (spectrograde) with and tetra-*n*-butylammonium hexafluorophosphate ([*n*-Bu_4N][PF_6]), 0.1 M as supporting electrolyte, both purchased from Sigma-Aldrich. The experiments were carried out in a conventional three-electrode cell at 20–21 °C with a platinum-disk working electrode (A = 0.070 cm^2), a Pt wire as auxiliary electrode and a saturated Calomel (SCE) as reference electrode. Solutions for cyclic voltammetry were typically 10^{-4} mM and the solutions were previously deoxygenated by purging with nitrogen.

The measurements in aqueous medium were in phosphate buffer (pH = 7.0)/ $NaClO_4$ 0.1 M. supporting electrolyte.

The surface coverages (G) were estimated from the electroactive ferrocenyl sites in the films from the integrated charge, *Q*, of the cyclic voltammetric waves.

Preparation of modified electrodes

The Pt disk electrodes were firstly polished using 0.1 μm of alumina powder and rinsed in ultrapure water in an ultrasonic bath. Each polished electrode was pre-conditioned by cycling in 0.5 M H_2SO_4 until a stationary cyclic voltammogram was obtained. Finally, the electrodes were rinsed successively with water and acetone and allowed to dry at room temperature. The films were deposited on the Pt electrodes (vs. SCE) from an electrolyte bath containing approximately 10^{-4} M Fc in 0.1 M tetra-*n*-butylammonium hexafluorophosphate (TBAH)/dichloromethane.

Electrode of **G1-DDSQ-Fc$_8$** was prepared by the controlled- potential electrolysis at 1 V for 5 min (**G1-DDSQ-Fc$_8$**-potentiostatic).
Electrodes of **G2-DDSQ-Fc$_{16}$** were prepared as follows:

- the first one by repeated cycling (20 cycle) between 0.0 to 1.0 V (**G2-DDSQ-Fc$_{16}$**-potentiodynamic).
- the second one was busked *via* controlled-potential electrolysis at 1.0 V for 5 min (**G2-DDSQ-Fc$_{16}$**-potentiostatic).

The modified electrodes were rinsed with dichloromethane and allowed to dry at room temperature. The surface coverage of electroactive ferrocenyl sites in the film (Γ) was determined from the integrated charge of the cyclic voltammetric waves.

Electrochemical Impedance Spectroscopy (EIS)

Electrochemical impedance spectroscopy measurements were carried out at 0.2 V *vs.* Ag/AgCl electrode over the frequency range of 0.1 to 10,000 Hz with 10 mV AC perturbation in 10 mM $K_3Fe(CN)_6/K_4Fe(CN)_6$ (1:1) containing 0.1 M KCl.

Scanning electron microscopy

Scanning Electron Microscopy (SEM) images were taken using an FEI Quanta 250 FEG microscope equipped with an EDAX EDS or vCD detector. Images were taken in high vacuum mode, and accelerating voltage (5 kV). Thin films of samples were fabricated on Pt wires using cyclic voltammetry technique: controlled-potential electrolysis at E= 1.0 V for 5 min for **G1-DDSQ-Fc$_8$** and repeated cycling (20 cycle) between 0.0 to 1.0 V for **G2-DDSQ-Fc$_{16}$**.

Thermogravimetric Analysis (TGA)

The analyses were performed using a TGA/DSC 1 Mettler-Toledo thermal gravimetric apparatus. The analyses were performed in an nitrogen atmosphere (flow of 60 mL/min), from ambient temperature to 700 °C at the heating rate of 10 °C/min. The temperature of initial degradation ($T^{5\%}$ and $T^{10\%}$) was taken as the onset temperature at which 5 wt% and 10 wt% of mass loss occurs, respectively.

3. Synthetic procedures

General synthetic procedure for the ferrocene-substituted silsesquioxanes obtained *via* hydrosilylation - exemplary reaction for the synthesis of G1-DDSQ-Fc$_8$

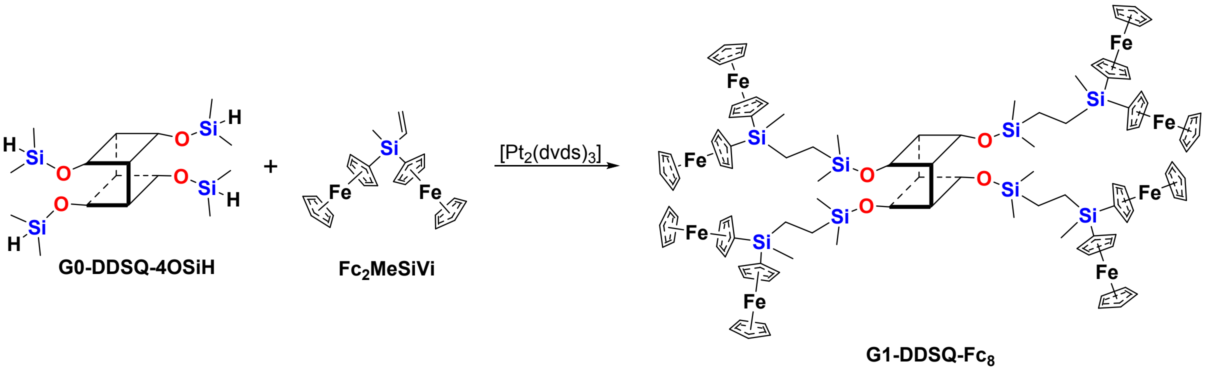

The synthetic protocol is presented for **G1-DDSQ-Fc$_8$** as an example. To a two-necked round-bottom flask equipped with a condenser and magnetic stirrer, **G0-DDSQ-4OSiH** (0.102 g, 0.08 mmol), toluene (3 mL) and **Fc$_2$MeSiVi** (0.166 g, 0.38 mmol) were placed in an argon atmosphere. The reaction was heated to 40 ºC and [Pt$_2$(dvds)$_3$] (3.58 µL, 0.31×10^{-3} mmol) was added. The reaction mixture was kept at 95 °C for 24 h. After cooling it to room temperature, the reaction mixture was transferred to a flask and evaporated under reduced pressure. The crude product was dissolved in DCM and transferred onto a dry chromatographic column (silica gel 60) and DCM was allowed to evaporate overnight. Next, the unreacted **Fc$_2$MeSiVi** was washed out with *n*-hexane:THF (80:1) as a eluent. After that, the **G1-DDSQ-Fc$_8$** was eluted with *n*-hexane:DCM (2:3). Evaporation of eluent gave an analytically pure sample of **G1-DDSQ-Fc$_8$** in 74% yield.

4. Additional spectra

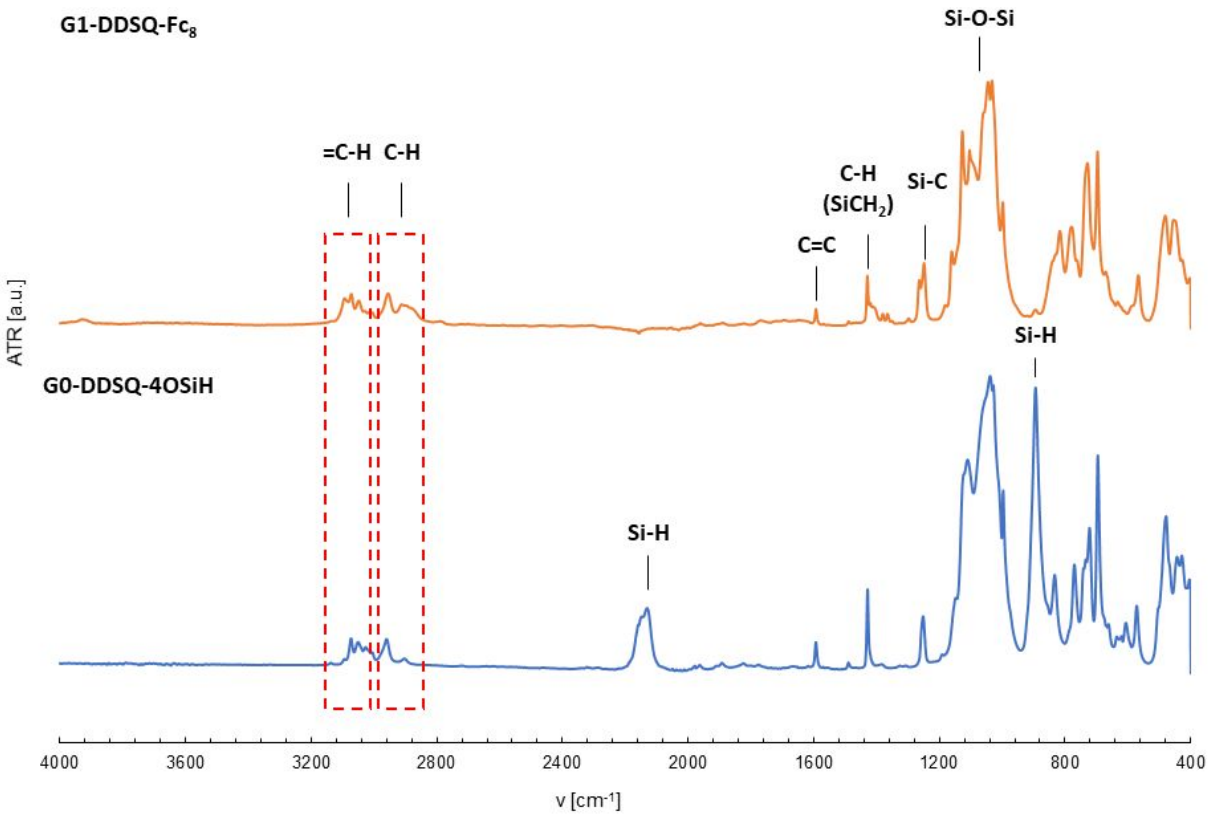

Figure S1 FT-IR stacked spectra of **G0-DDSQ-4OSiH** (blue) and **G1-DDSQ-Fc$_8$** (orange) for isolated compounds.

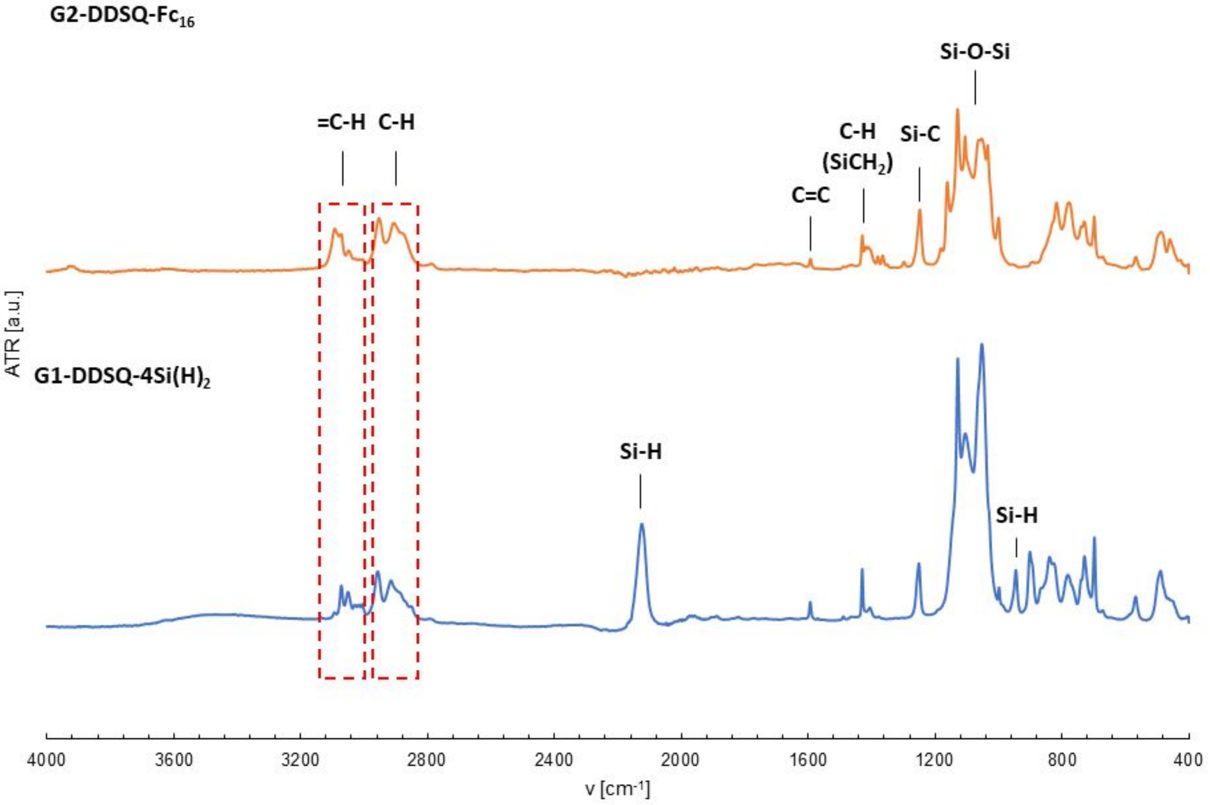

Figure S2 FT-IR stacked spectra of **G1-DDSQ-4Si(H)$_2$** (blue) and **G2-DDSQ-Fc$_{16}$** (orange) for isolated compounds.

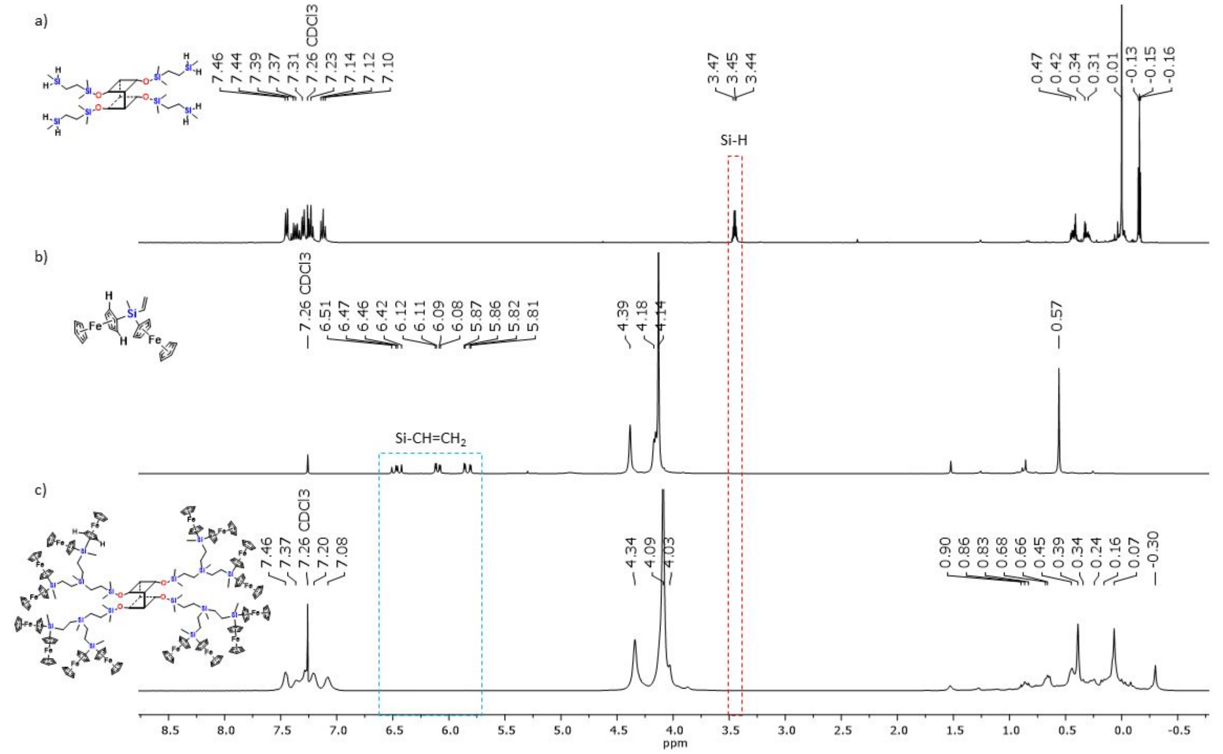

Figure S3 A selected range of stacked ^{1}H NMR spectra of a) **G1-DDSQ-4Si(H)$_2$**, b) **Fc$_2$MeSiVi**, c) **G2-DDSQ-Fc$_{16}$**.

5. Table of isolated compounds

Structure	Compound Abbrev.	Isolation yield [%]	Page
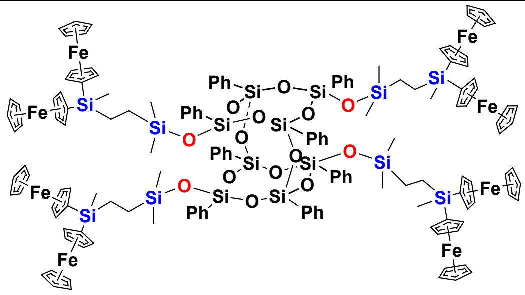	**G1-DDSQ-Fc$_8$**	74	**S-8-**
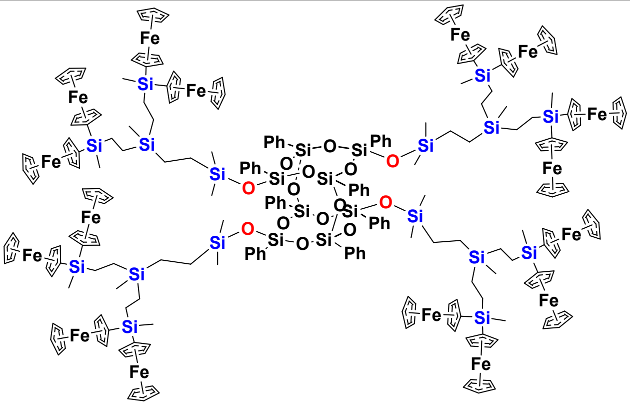	**G2-DDSQ-Fc$_{16}$**	60	**S-10-**

6. Characterization data of the obtained products (^{1}H, ^{13}C, ^{29}Si NMR spectra and IR spectroscopy)

G1-DDSQ-Fc$_8$

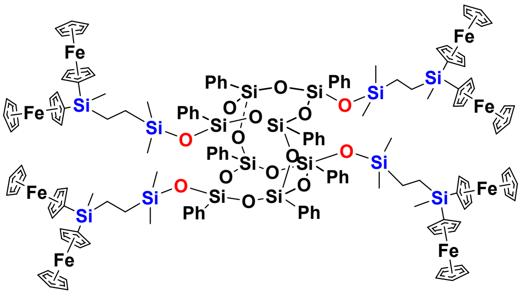

Orange solid. Isolated Yield 74%.

^{1}H NMR (400 MHz, $CDCl_3$, ppm): δ = 0.08 (m, 24H, -$SiCH_3$), 0.19 (s, 12H, -$SiCH_3$), 0.46-0.90 (m, 16H, -CH_2-), 3.90 (s, 10H, -Fc), 3.97 (s, 46H, -Fc), 4.26 (s, 16H, -Fc), 7.11-7.13 (m, 8H, Ph), 7.21 (t, J_{H-H}= 7.4 Hz, 8H, Ph), 7.32 (d, J_{H-H}= 7.0 Hz, 8H, Ph), 7.36-7.38 (m, 8H, Ph), 7.48 (d, J_{H-H}= 7.0 Hz, 8H, Ph).
^{13}C NMR (101 MHz, $CDCl_3$, ppm): δ = -3.16 (Si-CH_3), -0.23 (Si-CH_3), 7.67 (Si-CH_2), 10.65 (Si-CH_2), 68.46 (-Fc), 70.63-70.78 (-Fc), 73.68 (-Fc), 127.71-127.74 (Ph), 129.90 (Ph), 130.26 (Ph), 131.84 (Ph), 133.68 (Ph), 134.37-134.48 (Ph).
^{29}Si NMR (79.5 MHz, $CDCl_3$, ppm): δ = 11.62 (-Si-O), -4.76 (-Si-Fc), -75.99, -78.61 (-Si-Ph).
IR (ATR, cm^{-1}): 3093.54, 3072.39 (C-H), 3048.84 (C-H phenyl), 2955.29, 2911.64 (C-H), 1593.08, 1429.39 (C=C phenyl), 1264.43, 1248.94 (Si-C), 1160.79, 1127.13, 1104.00, 1045.33, 1032.74 (Si-O-Si), 998.17 (C-H phenyl).
EA: Anal. calcd for $C_{34}H_{72}O_{13}Si_9$ (%):C, 58.03; H, 5.40; found: C, 58.26; H, 5.42.
MALDI-TOF MS: Calcd. for $C_{148}H_{166}Fe_8O_{14}Si_{16}^{2+}$: m/z 3062.3370 [M + 2H]$^{2+}$. Found: 3062.3106.

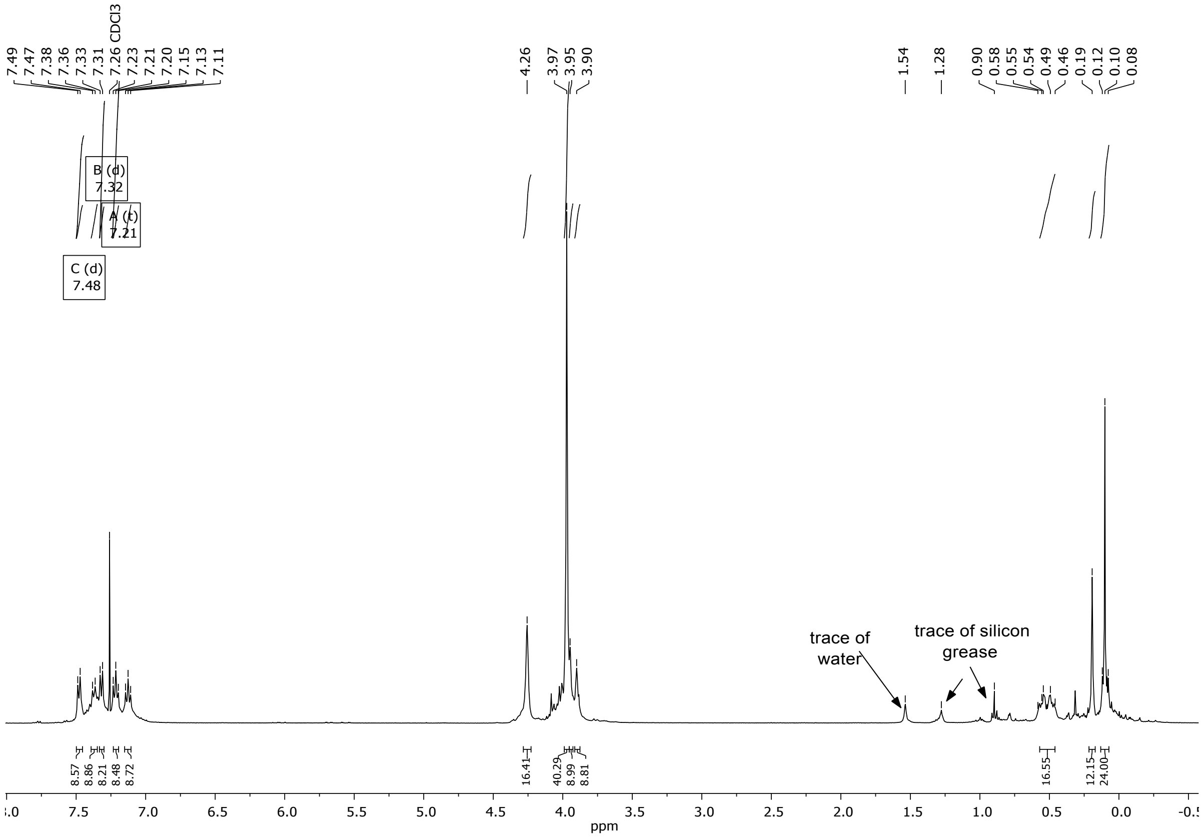

Figure S4 ^{1}H NMR (400 MHz, $CDCl_3$) spectrum of **G1-DDSQ-Fc$_8$**.

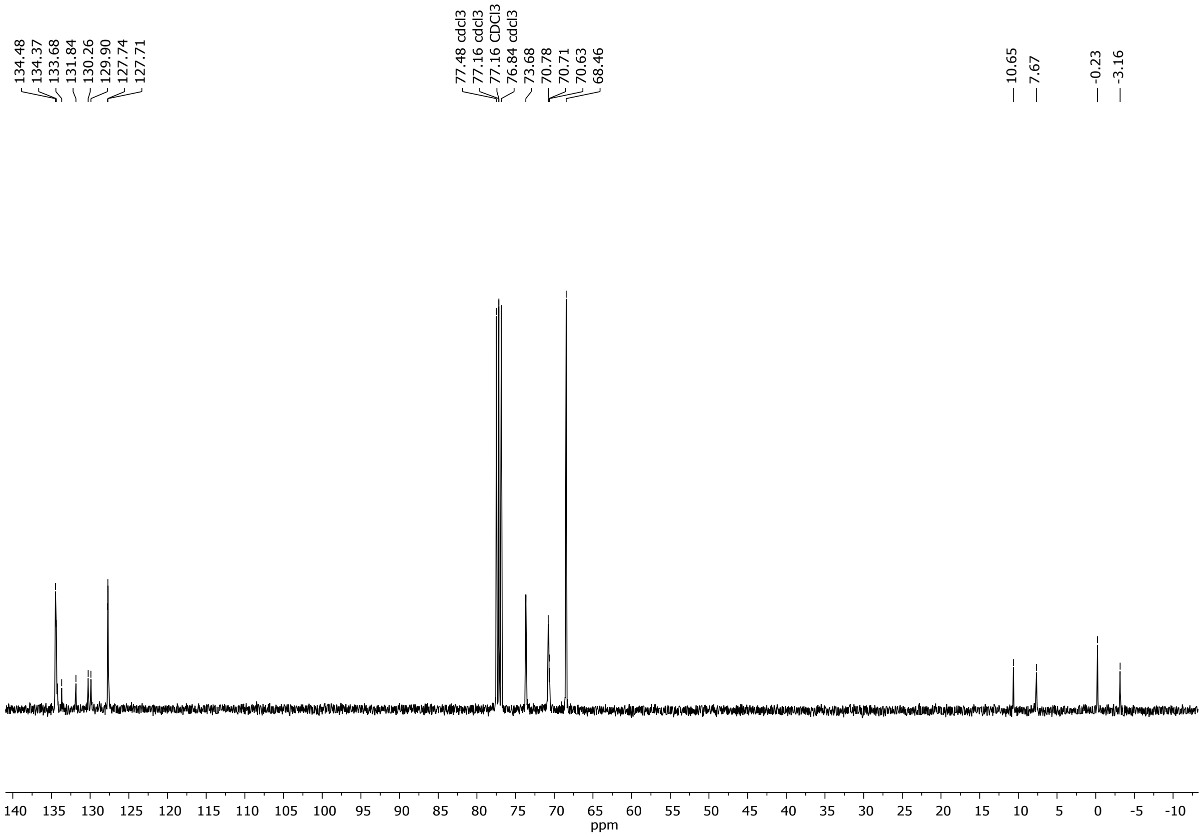

Figure S5 ^{13}C NMR (101 MHz, $CDCl_3$) spectrum of **G1-DDSQ-Fc$_8$**.

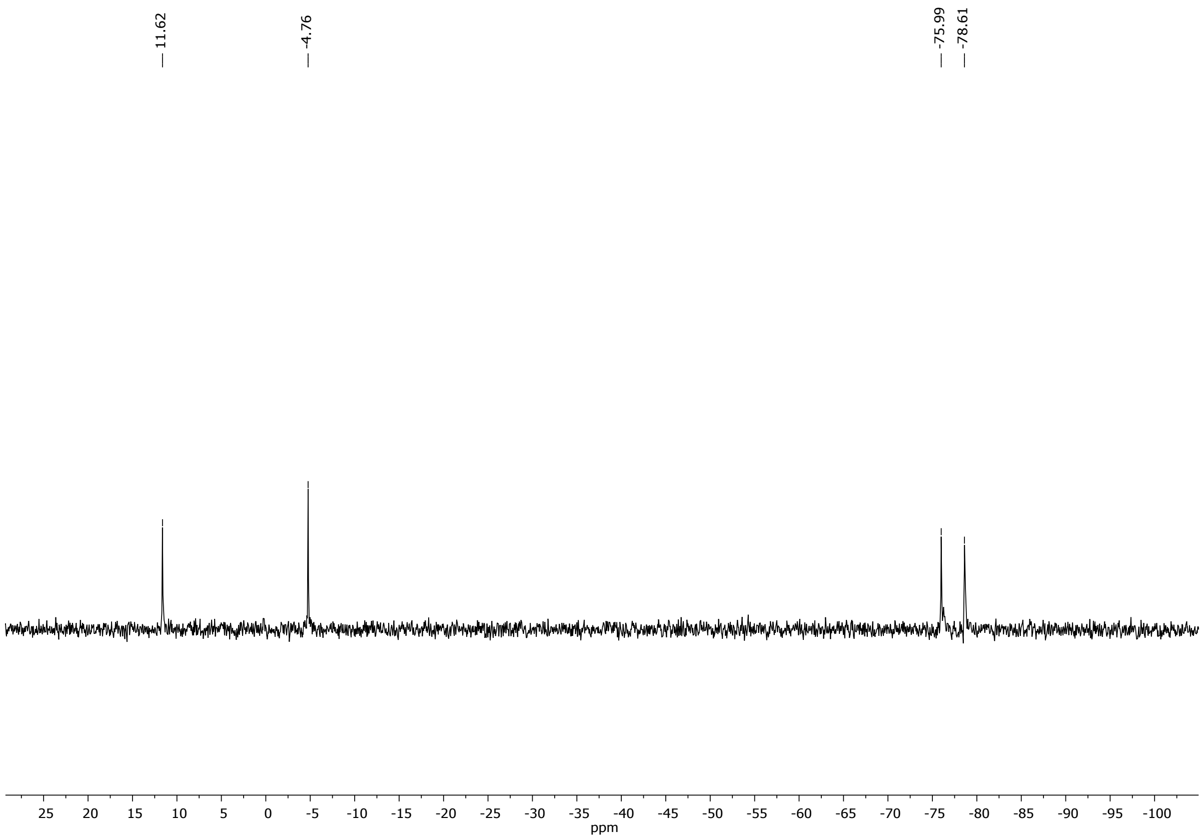

Figure S6 ^{29}Si NMR (79.5 MHz, $CDCl_3$) spectrum of **G1-DDSQ-Fc$_8$**.

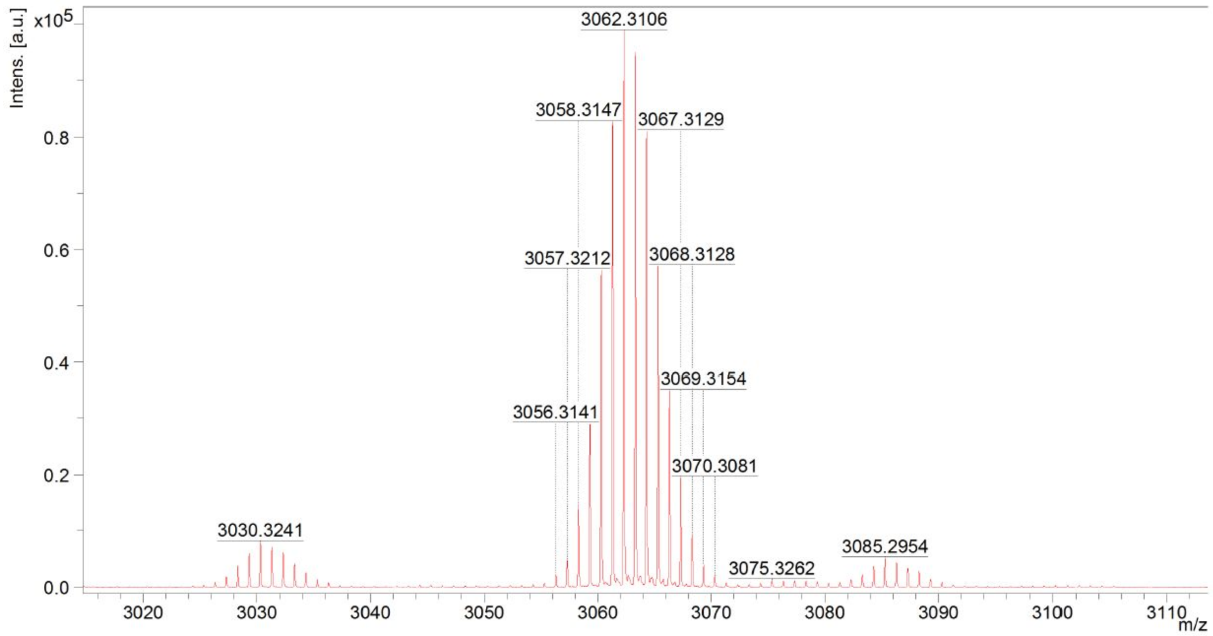

Figure S7 MALDI-TOF-MS spectrum of **G1-DDSQ-Fc$_8$**.

G2-DDSQ-Fc$_{16}$

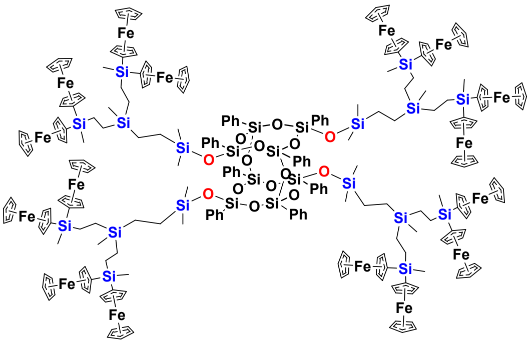

Orange solid. Isolated Yield 60%.

^{1}H NMR (400 MHz, $CDCl_3$, ppm): δ = -0.30 (s, 12H, -$SiCH_3$), 0.07 (s, 24H, -$SiCH_3$), 0.16-0.18 (m, 8H, -CH_2-), 0.24-0.28 (m, 8H, -CH_2-), 0,39 (s, 24h, -$SiCH_3$), 0.45 (s, 8H, -CH_2-), 0.64-0.68 (m, 16H, -CH_2-), 0.84-0.90 (m, 8H, -CH_2-), 4.06 (d, $J_{H\text{-}H}$ = 24.2 Hz, 112H, -Fc), 4.34 (s, 32H, -Fc), 7.08 (s, 8H, Ph), 7.21 (s, 8H, Ph), 7.29 (s, 8H, Ph), 7.37 (s, 8H, Ph), 7.46 (s, 8H, Ph).
^{13}C NMR (101 MHz, $CDCl_3$, ppm): δ = -6.79 (Si-CH_3), -3.07 (Si-CH_3), -0.21 (Si-CH_3), 4.16 (Si-CH_2), 5.05 (Si-CH_2), 8.46 (Si-CH_2), 10.26 (Si-CH_2), 68.28-68.60 (-Fc), 70.82-70.97 (-Fc), 73.76 (-Fc), 127.62 (Ph), 129.86, 130.21 (Ph), 131.85 (Ph), 133.67 (Ph), 134.33-134.44 (Ph).
^{29}Si NMR (79.5 MHz, $CDCl_3$, ppm): δ = 11.54 (-Si-O), 8.04 (-Si-CH_2), 1.47 (-Si-Fc), -4.68 (-Si-Fc), -76.04, -78.67 (-Si-Ph).
IR (ATR, cm^{-1}): 3093.17, 3072.71 (C-H), 3048.61 (C-H phenyl), 2953.01, 2906.28 (C-H), 1593.05, 1429.41 (C=C phenyl), 1264.45, 1247.79 (Si-C), 1160.60, 1127.65, 1104.36, 1047.14, 1032.70 (Si-O-Si), 998.62 (C-H phenyl).
EA: Anal. calcd for $C_{252}H_{292}O_{14}Si_{24}$ (%): C- 59.20, H- 5.76; found: C- 59.44; H- 5.78.
MALDI-TOF MS: Calcd. for $C_{252}H_{296}Fe_{16}O_{14}Si_{24}^{4+}$: m/z 5112.6481 [M + 4H]$^{4+}$. Found: 5112.5801.

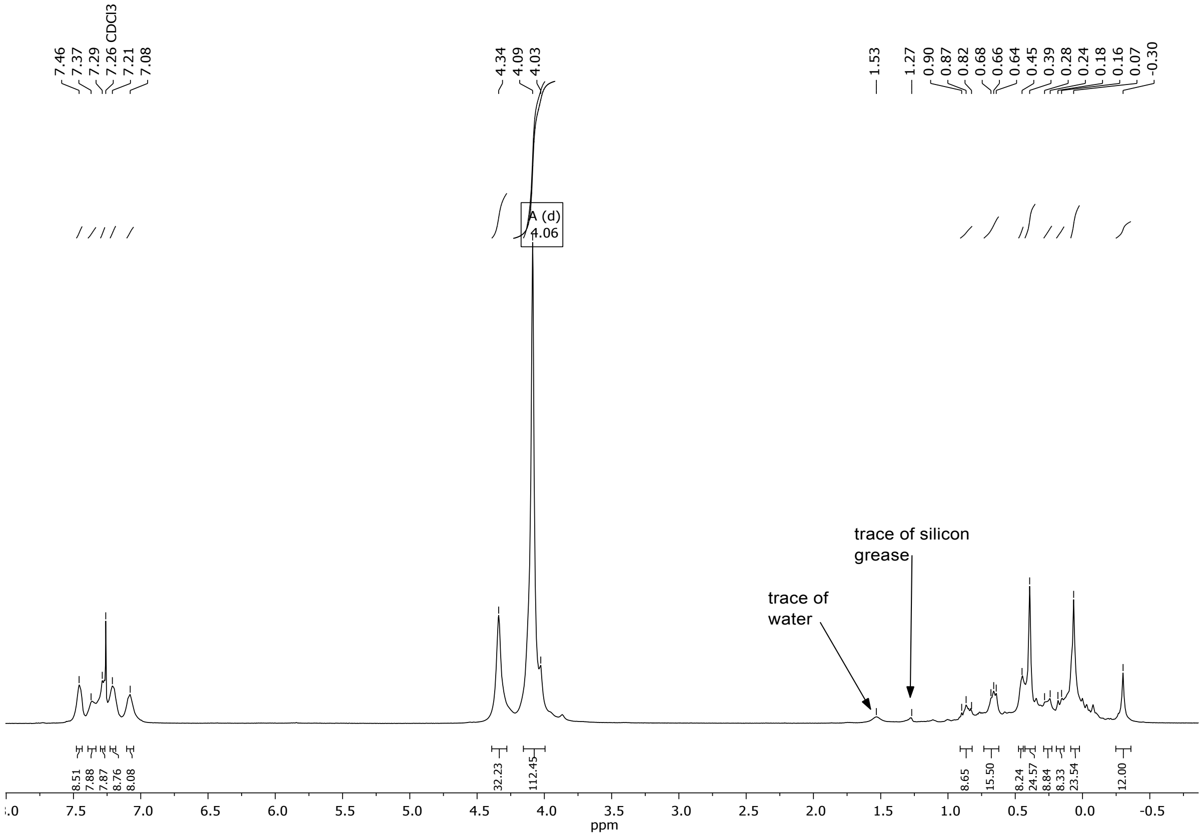

Figure S8 ^{1}H NMR (400 MHz, $CDCl_3$) spectrum of **G2-DDSQ-Fc$_{16}$**.

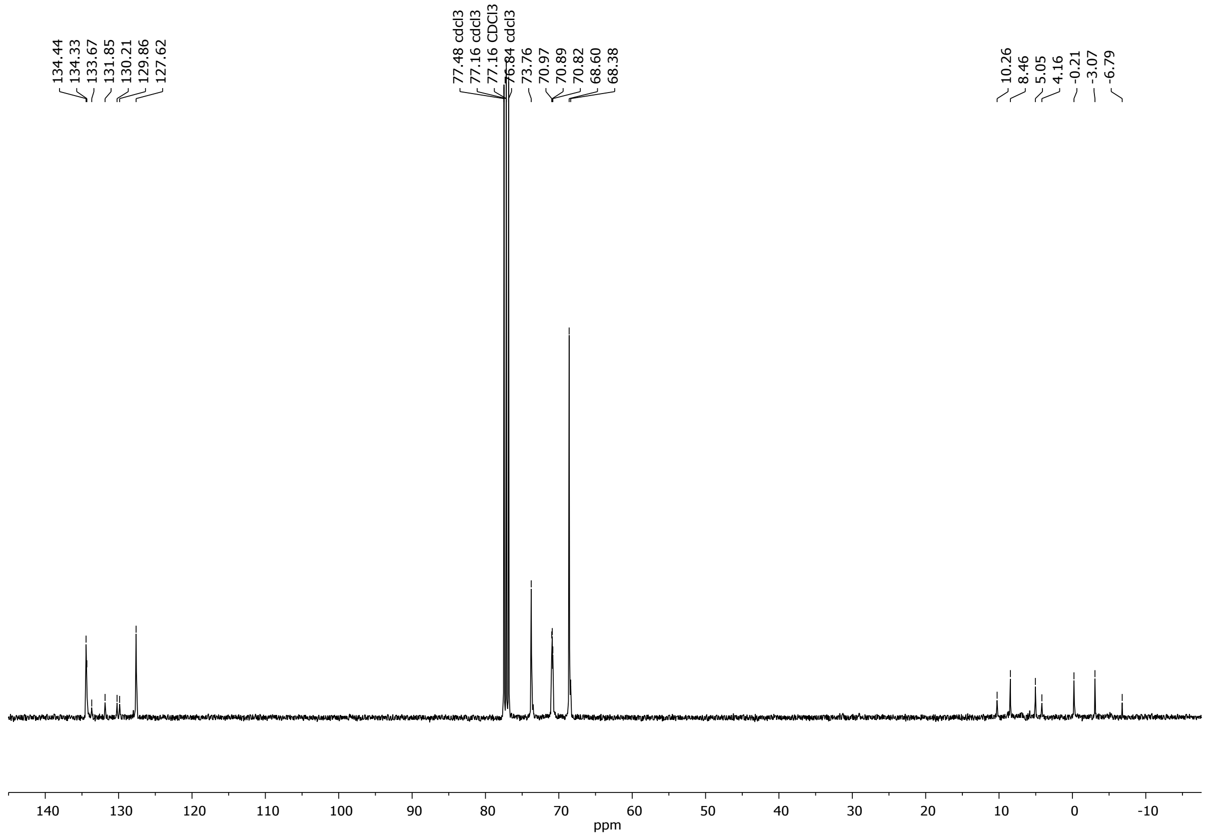

Figure S9 ^{13}C NMR (101 MHz, $CDCl_3$) spectrum of **G2-DDSQ-Fc$_{16}$**.

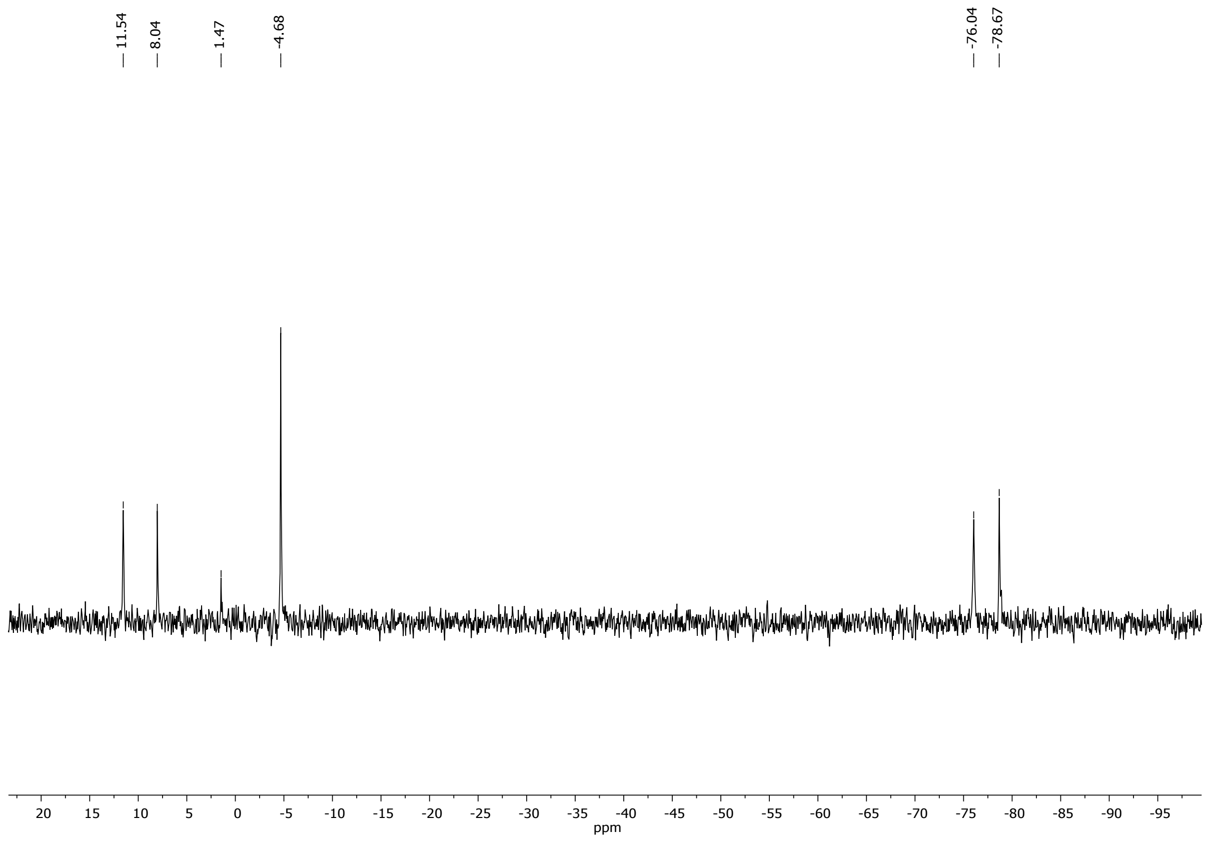

Figure S10 ^{29}Si NMR (79.5 MHz, $CDCl_3$) spectrum of **G2-DDSQ-Fc$_{16}$**.

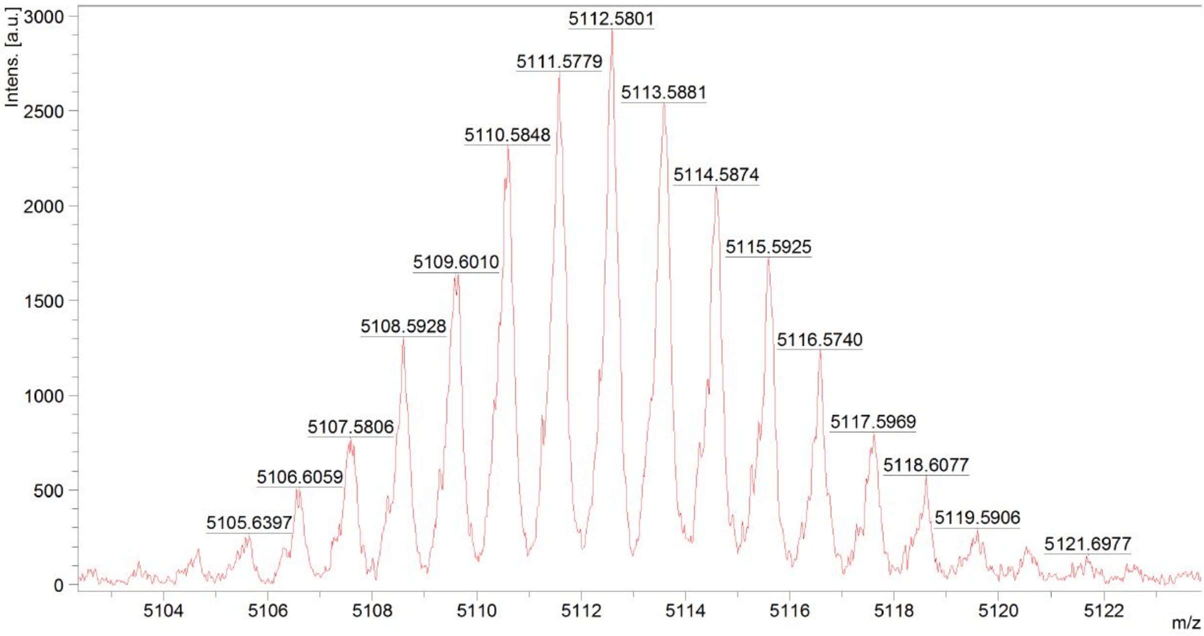

Figure S11 MALDI-TOF-MS spectrum of **G2-DDSQ-Fc$_{16}$**.

7. Solubility of obtained products

Table S1 The solubility of obtained systems in selected solvents.[a)]

	**G1-DDSQ-Fc$_8$**	**G2-DDSQ-Fc$_{16}$**
DCM	20 μL	30 μL
Et_2O	40 μL	60 μL
THF	50 μL	55 μL
Toluene	55 μL	55 μL
MeOH	insoluble*(solid)	insoluble* (oil)
MeCN	insoluble*	insoluble*
n-hexane	insoluble* (yellowish)	insoluble*

[a)] The solubility was checked for 20 mg samples; *sample was insoluble in 7 mL of solvent.

8. TGA analysis

Table S2 Thermal properties of obtained compounds measured in nitrogen.

**Prod. Abbreviation**	**Mass Loss Temperature [°C]**		**Residue at 700 °C [%]**
	$T_d^{5\%}$	$T_d^{10\%}$	
**G1-DDSQ-Fc$_8$**	431	455	61
**G2-DDSQ-Fc$_{16}$**	421	442	54

9. Cyclic Voltammetry studies

- **Electrochemical studies in solution**

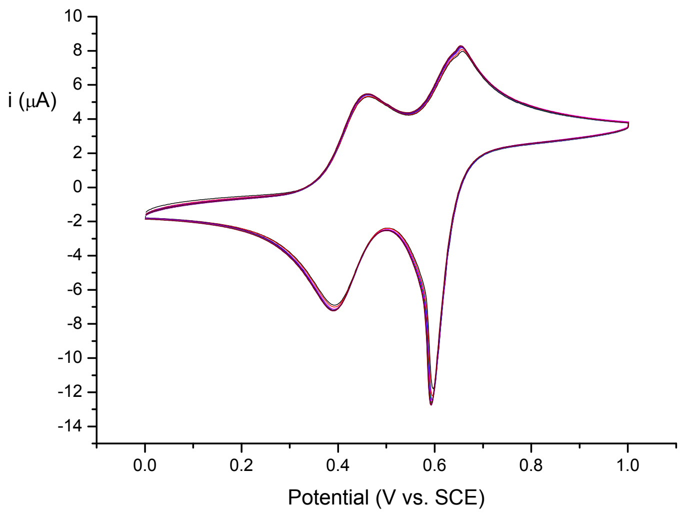

Figure S12 Successive cyclic voltammograms of dendrimer **G1-DDSQ-Fc$_8$** in CH_2Cl_2 solution with *n*-Bu_4NPF_6 0.1 M solution (scan rate: 100 mV s^{-1}).

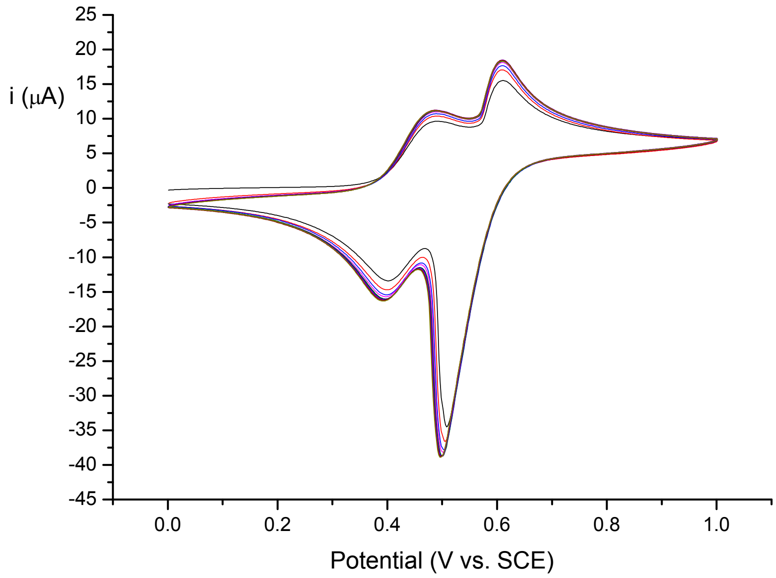

Figure S13 Successive cyclic voltammograms of dendrimers **G2-DDSQ-Fc$_{16}$** in CH_2Cl_2 with *n*-Bu_4NPF_6 0.1 M solution (scan rate: 100 mV s^{-1}).

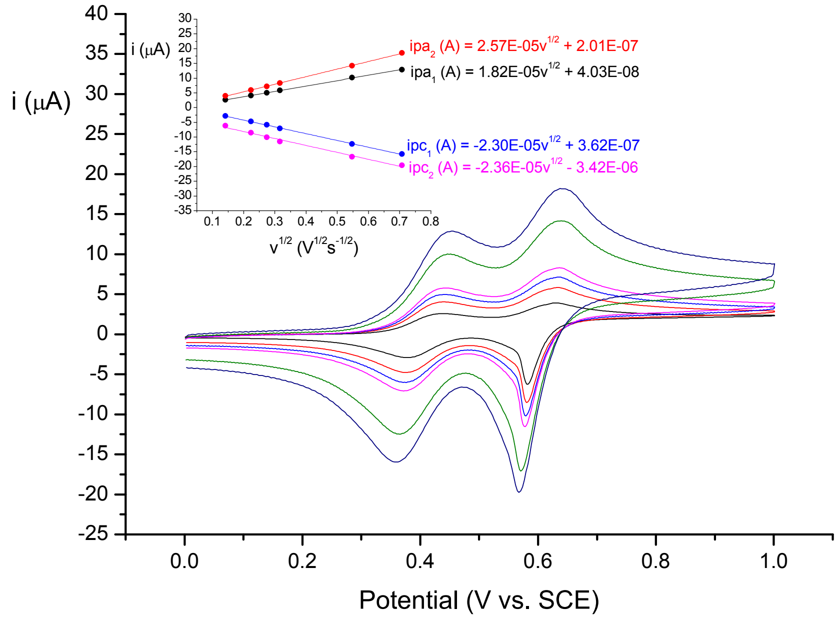

Figure S14 Increase of peak current with the successive increasing scan rate in CH_2Cl_2 solution of **G1-DDSQ-Fc$_8$**. Scan rates: 20, 50, 75, 100, 300 and 500 mV s^{-1}. Inset: linear dependence of the peak currents on the square root of the scan rate.

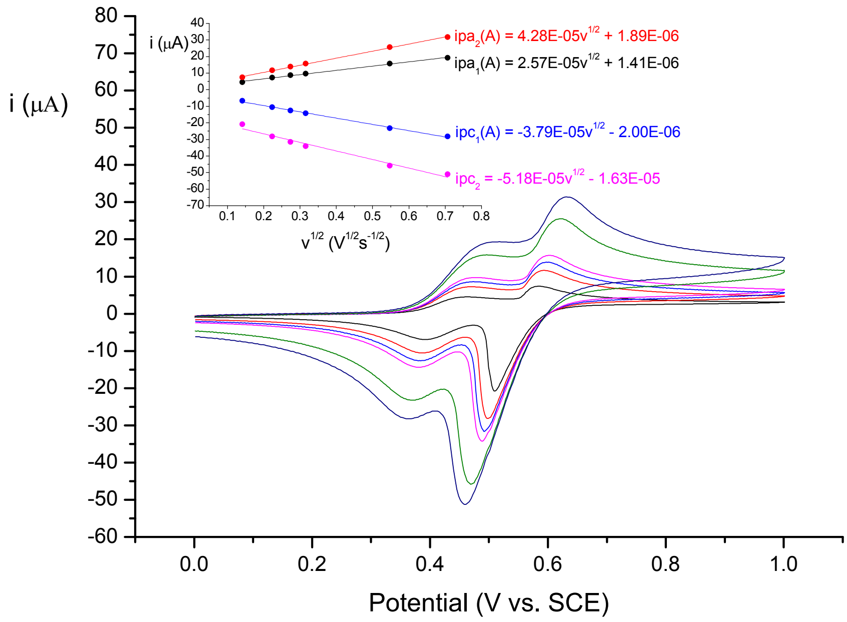

Figure S15 Increase of peak current with the successive increasing scan rate in CH_2Cl_2 solution of **G2-DDSQ-Fc$_{16}$**. Scan rates: 20, 50, 75, 100, 300 and 500 mV s^{-1}. Inset: linear dependence of the peak currents on the square root of the scan rate.

The comproportionation constants (K_c) calculations:

G1-DDSQ-Fc$_8$:

$$E_1^0 = \frac{E_{pa1} + E_{pc1}}{2} = \frac{0.44 + 0.38}{2} = 0.41\ V \quad \text{(S1)}$$

$$E_2^0 = \frac{E_{pa2} + E_{pc2}}{2} = \frac{0.63 + 0.58}{2} = 0.61\ V \quad \text{(S2)}$$

$$\Delta E_{p2-1}^0 = 0.61 - 0.41 = 0.20\ V \quad \text{(S3)}$$

$$K_c = \frac{[B]^{n_1+n_2}}{[C]^{n_1}[A]^{n_2}} = exp\left[\frac{(E_2^0 - E_1^0)n_1 n_2 F}{RT}\right] = \exp\left[\frac{\Delta E^0}{25{,}69}\right] = \exp\left[\frac{200mV}{25{,}69}\right] = 2405 \quad \text{(S4)}$$

G2-DDSQ-Fc$_{16}$:

$$E_1^0 = \frac{E_{pa1} + E_{pc1}}{2} = \frac{0.46 + 0.39}{2} = 0.42\ V \quad \text{(S5)}$$

$$E_2^0 = \frac{E_{pa2} + E_{pc2}}{2} = \frac{0.59 + 0.51}{2} = 0.55\ V \quad \text{(S6)}$$

$$\Delta E_{p2-1}^0 = 0.55 - 0.42 = 0.13\ V \quad \text{(S7)}$$

$$K_c = \frac{[B]^{n_1+n_2}}{[C]^{n_1}[A]^{n_2}} = exp\left[\frac{(E_2^0 - E_1^0)n_1 n_2 F}{RT}\right] = \exp\left[\frac{\Delta E^0}{25{,}69}\right] = \exp\left[\frac{130mV}{25{,}69}\right] = 157{,}6 = 158 \quad \text{(S8)}$$

A)

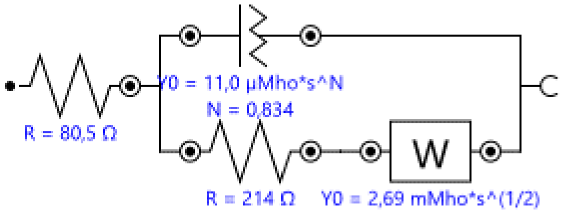

B)

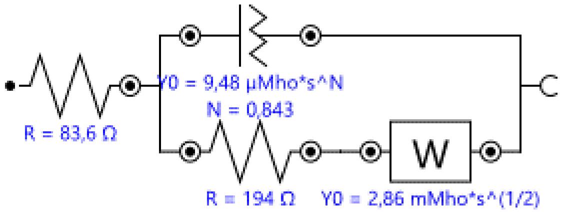

C)

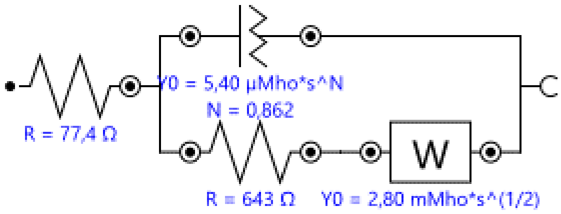

Figure S16 Equivalent circuits obtained from fit and simulation of the A) potentiostatic **G1-DDSQ-Fc$_8$**, B) potentiodynamic **G2-DDSQ-Fc$_{16}$**, and C) potentiostatic **G2-DDSQ-Fc$_{16}$** modified electrodes EIS data.

10. SEM images

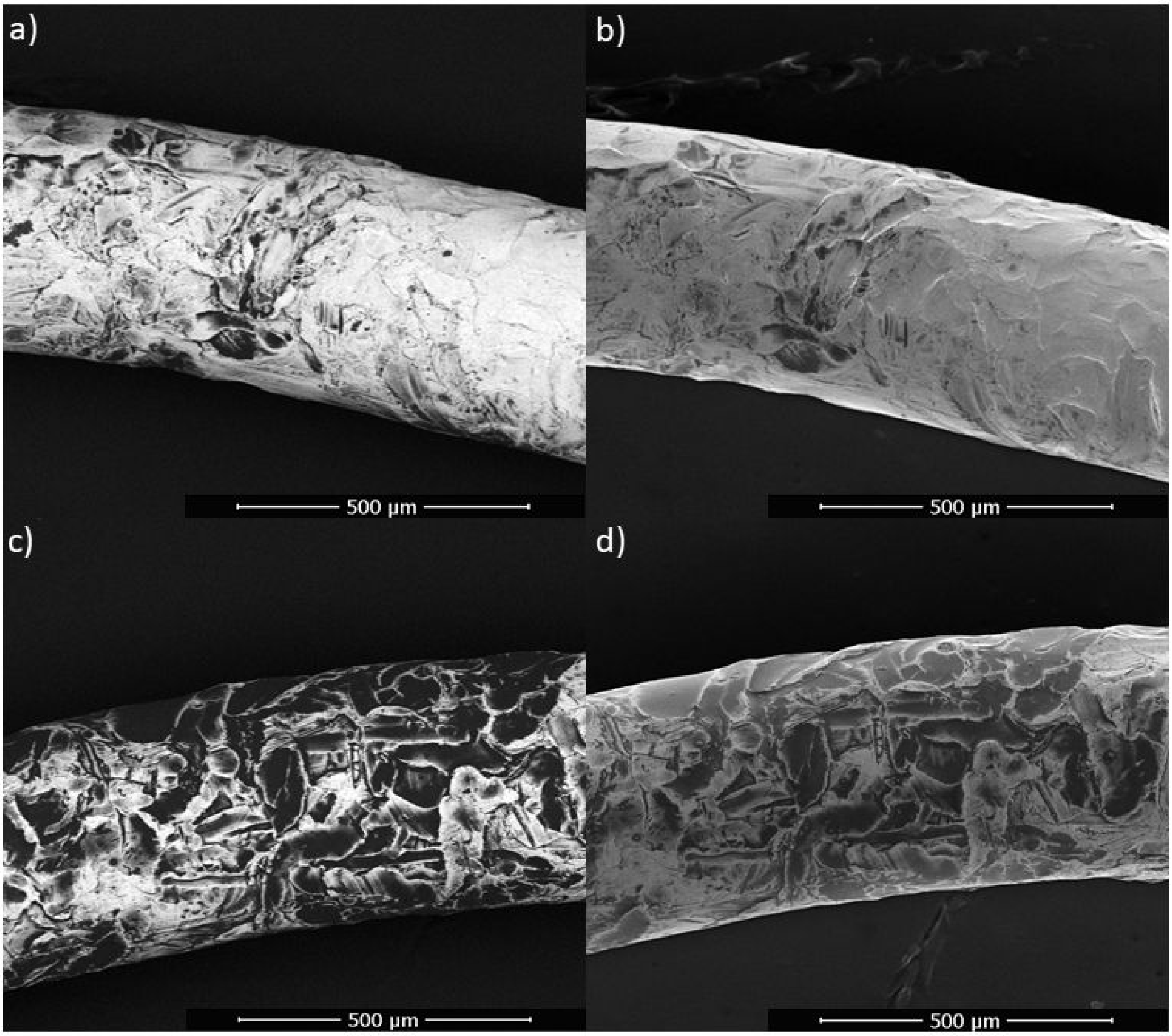

Figure S17 Scanning Electron Microscopy images of Pt wires modified by **G2-DDSQ-Fc$_{16}$** with using repeated cycling method (20 cycle) between 0.0 to 1.0 V: a) and c) BSE (BackScattered Electrons) images; b) and d) SE (Secondary Electrons) detector.

12. References:

(1) Cuadrado, I.; Casado, C. M.; Alonso, B.; Moran, M.; Losada, J.; Belsky, V. Dendrimers Containing Organometallic Moieties Electronically Communicated. *J. Am. Chem. Soc.* **1997**, *119* (32), 7613–7614.

(2) Mituła, K.; Duszczak, J.; Brząkalski, D.; Dudziec, B.; Kubicki, M.; Marciniec, B. Tetra-Functional Double-Decker Silsesquioxanes as Anchors for Reactive Functional Groups and Potential Synthons for Hybrid Materials. *Chem. Commun.* **2017**, *53* (75), 10370–10373.

(3) Mrzygłód, A.; Januszewski, R.; Duszczak, J.; Dutkiewicz, M.; Kubicki, M.; Dudziec, B. Tricky but Repeatable Synthetic Approach to Branched, Multifunctional Silsesquioxane Dendrimer Derivatives. *Inorg. Chem. Front.* **2023**, *10*, 4587–4596.
